# SpyKing—Privacy-preserving framework for Spiking Neural Networks

**DOI:** 10.3389/fnins.2025.1551143

**Published:** 2025-05-30

**Authors:** Farzad Nikfam, Alberto Marchisio, Maurizio Martina, Muhammad Shafique

**Affiliations:** ^1^Very Large Scale Integration Laboratory, Department of Electronics Engineering, Politecnico di Torino, Torino, Italy; ^2^eBrain Lab, Division of Engineering, New York University, Abu Dhabi, United Arab Emirates

**Keywords:** Deep Neural Network (DNN), Homomorphic Encryption (HE), LeNet5, machine learning, privacy-preserving, safety, security, Spiking Neural Network (SNN)

## Abstract

Artificial intelligence (AI) models, frequently built using deep neural networks (DNNs), have become integral to many aspects of modern life. However, the vast amount of data they process is not always secure, posing potential risks to privacy and safety. Fully Homomorphic Encryption (FHE) enables computations on encrypted data while preserving its confidentiality, making it a promising approach for privacy-preserving AI. This study evaluates the performance of FHE when applied to DNNs and compares it with Spiking Neural Networks (SNNs), which more closely resemble biological neurons and, under certain conditions, may achieve superior results. Using the SpyKing framework, we analyze key challenges in encrypted neural computations, particularly the limitations of FHE in handling non-linear operations. To ensure a comprehensive evaluation, we conducted experiments on the MNIST, FashionMNIST, and CIFAR10 datasets while systematically varying encryption parameters to optimize SNN performance. Our results show that FHE significantly increases computational costs but remains viable in terms of accuracy and data security. Furthermore, SNNs achieved up to 35% higher absolute accuracy than DNNs on encrypted data with low values of the plaintext modulus *t*. These findings highlight the potential of SNNs in privacy-preserving AI and underscore the growing need for secure yet efficient neural computing solutions.

## 1 Introduction

Recent research (Nikfam et al., 2023) has explored the comparison between Deep Neural Networks (DNNs) and Spiking Neural Networks (SNNs) in the context of Fully Homomorphic Encryption (FHE), a powerful cryptographic technique that enables computation on encrypted data without decryption. While encryption is crucial for privacy-preserving (Barni et al., 2006; Chabanne et al., 2017; Disabato et al., 2020), FHE introduces a significant computational overhead, making conventional DNNs less efficient. In this work, we investigate whether SNNs, known for their sparse and energy-efficient processing, can offer a viable alternative to DNNs under FHE constraints. Given their event-driven nature, SNNs require fewer operations and may alleviate some of the computational burden imposed by FHE. Moreover, integrating SNNs with FHE enables secure neural network inference on sensitive data, with potential applications in healthcare (e.g., encrypted medical image analysis), finance (e.g., fraud detection on encrypted transactions), and cybersecurity. By comparing the performance of LeNet5-based SNN (von Kügelgen, 2017) and DNN (Schmidhuber, 2015) models on both encrypted and plaintext data, we aim to highlight the advantages and limitations of using SNNs for privacy-preserving. Our findings indicate that while FHE remains computationally expensive, SNNs (Kim et al., 2022) can, under certain conditions, outperform DNNs, making them a promising candidate for secure and efficient encrypted neural computation (Gilad-Bachrach et al., 2016).

**Figure 1:**
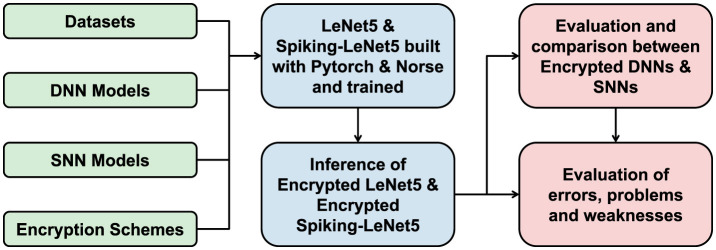
A summary flowchart of the SpyKing research project.

Figure 1 shows a summary diagram of the research work carried out for SpyKing, from the inputs used to the results obtained.

## 2 Spiking Neural Network

**Figure 2:**
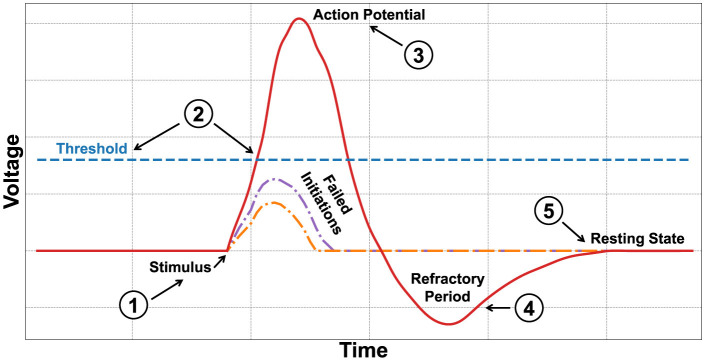
An example of a spiking neuron that activates only after receiving the necessary charge to surpass the threshold, undergoing a refractory period before returning to a resting state.

A SNN (von Kügelgen, 2017; Ponulak and Kasiński, 2011), or pulse neural network, is a type of neural architecture inspired by the functioning of biological neurons in the brain. Unlike traditional neural networks, such as DNNs, SNNs use a communication model based on pulses or spikes, representing signals sent by neurons (see Figure 2).

In traditional models, artificial neurons, after receiving input, apply a transformation using an activation function and produce a continuous output. In spiking neurons (Izhikevich, 2003), communication occurs through discrete pulses or *spikes* (Roy et al., 2019). Each neuron accumulates input signals (see pointer ①–Figure 2) over time and generates a spike when a certain threshold is exceeded (see pointer ②–Figure 2). Synapses, the connections between neurons, are determined by weights that can change during the learning process, increasing or decreasing the probability of a neuron firing. The activation of each neuron is based on both spatial and temporal factors. Each neuron depends on its position and connections with nearby neurons, and its activation is influenced by the time of charge before firing, which typically cannot be less than a certain threshold. When a neuron releases a spike (see pointer ③–Figure 2) after its potential reaches the threshold, its charge is reset, and the neuron enters a passive waiting phase, the refractory period (see pointer ④–Figure 2) before the resting state (see pointer ⑤–Figure 2).

This construction allows SNNs to closely mimic the real and biological functioning of the human brain. Considering the latency times between spikes due to charge times, SNNs also enable more energy-efficient models (Paugam-Moisy and Bohté, 2012).

### 2.1 Leaky Integrate-and-Fire

In the context of SNNs, Leaky Integrate-and-Fire (LIF) is a specific type of spiking neuron model. To better understand how it works, here's an explanation of the acronym LIF:

- Integration–the LIF neuron accumulates input over time. Each time it receives a spike, its *charge* increases. This accumulation of charge represents how the neuron *integrates* information over time.
- Firing - when the neuron's charge reaches a certain threshold, the neuron *fires* a spike. This simulates the idea of activation in the context of neural networks.
- Leak - the *leak* indicates that, over time, the neuron's charge tends to dissipate or lose energy. This process of charge loss over time is implemented to simulate the dynamic and adaptive nature of biological neurons.

So, the LIF model is essentially a way to describe how a spiking neuron accumulates and releases energy over time, reflecting some features of biological neurons. Its simplicity makes it computationally efficient, and the addition of the leak component makes it more adaptable and realistic compared to some more basic spiking neuron models.

There are other SNN neuron models, such as Hodgkin-Huxley (Amirsoleimani et al., 2016), which are based on very complex differential calculations, making it challenging to construct large computational models due to lower efficiency. Considering the trade-off between efficiency and reliability, the LIF neuron model was chosen for the creation of SpyKing.

### 2.2 Norse library

Norse (Pehle and Pedersen, 2021) is a Python (Raschka et al., 2020) library that leverages the advantages of bio-inspired neural components. Norse extends the PyTorch (Paszke et al., 2019) library for implementing DNN with primitives for biologically inspired neural components.

With Norse, it is possible to start with basic PyTorch DNN models and create their spiking versions. As we will see later in this work, the Lenet5 model, implemented in PyTorch, was used, and with the use of Norse, the spiking version, Spiking-Lenet5 (Han and Roy, 2020; Lee et al., 2016, 2019; Zenke and Ganguli, 2018; Tavanaei et al., 2019), was created.

#### 2.2.1 LIF parameters

The LIF parameters within Norse are specific configurations that define the behavior of LIF neurons in SNNs. These parameters include:

- $\tau_{syn}^{-1}$ - represents the inverse of the synaptic time constant, determining how quickly the synaptic input decays over time.
- $\tau_{mem}^{-1}$ - represents the inverse of the membrane time constant, influencing the rate of decay of the neuron's membrane potential without input.
- ***v*_*leak*_** - specifies the leak potential of the neuron, indicating the resting potential of the membrane when there is no synaptic input or other stimuli.
- ***v*_*th*_** - defines the threshold potential of the neuron. An action potential is generated when the membrane potential reaches or exceeds this threshold.
- ***v*_*reset*_** - represents the reset potential of the neuron. After firing an action potential, the membrane potential is reset to this value.

These parameters play a critical role in determining the dynamics of the LIF neuron in the SNN. They govern how the neuron integrates and responds to incoming synaptic input, as well as when it generates an action potential. The specific values of these parameters can be adjusted to achieve the desired behavior, providing control over the firing rate and responsiveness of the neuron within the network.

#### 2.2.2 Encoders

SNNs require an encoder to process temporal data represented as spikes. Since most ML datasets lack an inherent temporal structure, an encoding phase is essential to introduce the necessary temporal component. The encoder transforms input data into spike sequences, which are then processed by the SNN as tensors with binary values.

In a preliminary study phase (Casaburi et al., 2022), we compared the Constant Current LIF encoder, Poisson encoder, and Spike Latency LIF encoder. After conducting several analyses, we observed that the Constant Current LIF encoder yielded higher accuracy and, more importantly, allowed us to maintain a computationally efficient model for subsequent calculations. As a result, we selected it as the baseline for our experiments.

The Constant Current LIF encoder, implemented in the Norse library, is an encoding method that converts constant input into constant voltage spikes. Over a specified time interval, known as *seq*_*length*_, spikes are generated based on the input current. This approach enables Norse to operate on sparse input data as a sequence of binary tensors, optimizing the SNN's processing efficiency. If the potential reaches the required threshold during *seq*_*length*_, a spike is emitted.

In our encoding process, the spike threshold is static, meaning it remains constant throughout the simulations. This threshold, denoted as *v*_*th*_, defines the membrane potential level that must be reached for a neuron to emit a spike. Since the encoding phase relies on temporal sequences, the interplay between *v*_*th*_ and *seq*_*length*_ affects the spike generation rate. A lower *v*_*th*_ leads to more frequent spikes within *seq*_*length*_, whereas a higher threshold results in sparser spiking activity. This static threshold approach ensures a controlled and reproducible encoding process across all experiments.

Simply put, *seq*_*length*_ represents the number of iterations a SNN needs to biologically simulate the human brain. Consequently, the *seq*_*length*_ value serves as a temporal multiplier. For example, if a DNN takes time *x* to be trained or evaluate data, the corresponding SNN model will take a time equivalent to *x* multiplied by *seq*_*length*_. This poses a temporal efficiency challenge intrinsic to SNNs, and this temporal factor that elongates computation times cannot be eliminated. The only solution to address this issue is to choose a *seq*_*length*_ value that is balanced, accurately simulating SNNs without excessively extending computation times.

## 3 Homomorphic Encryption

**Figure 3:**
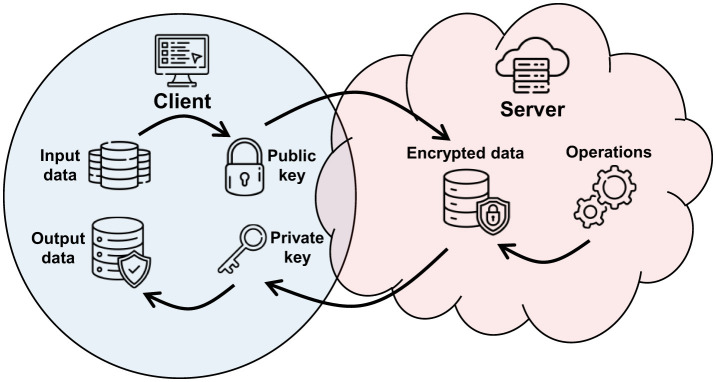
A HE scheme with a clear separation between client and server, where the data and results in plaintext are visible only to the client.

Homomorphic Encryption (HE) (Acar et al., 2018; Orlandi et al., 2007; Bos et al., 2013; Cammarota, 2022; Cousins et al., 2023) is an advanced form of cryptography that enables operations on encrypted data without the need for prior decryption. This technique is particularly useful when preserving data privacy (Falcetta and Roveri, 2022) during processing in environments where security is crucial, such as in cloud computing. Examining Figure 3 provides a clearer understanding of how HE works. Initial data is encrypted with a public key (Stehlé et al., 2009; Paillier, 1999) that anyone can obtain. Once encrypted, the data is sent to the server where it undergoes manipulation and specific computations. Finally, the results, still encrypted, are sent back to the client, who is the only entity capable of decrypting them using a secret key known only to them. In this manner, the entire data processing is kept secret, and only the client knows the original data and the final results.

The security of HE relies on the strength of the encryption algorithm and the secrecy of the keys. Unfortunately, there are limitations because computations on encrypted data are much more time, memory, and energy consuming, and therefore are only executed when necessary.

The term *homomorphic* indicates that operations performed on encrypted data correspond to the same operations executed on unencrypted data. Homomorphism can take various forms, including partially HE (Ryu et al., 2023), somewhat HE (Bonnoron et al., 2017) and fully HE (FHE) (Brakerski and Vaikuntanathan, 2014; Gentry, 2009a,b; Fan and Vercauteren, 2012; Brakerski et al., 2011). Each of these allows different levels of computation on encrypted data.

### 3.1 Fully Homomorphic Encryption

FHE (Brakerski and Vaikuntanathan, 2014; Gentry, 2009a,b; Fan and Vercauteren, 2012; Brakerski et al., 2011) is the most comprehensive form of HE, as it enables both addition and multiplication operations on encrypted data. One of the widely used schemes in this field is the Brakerski/Fan-Vercauteren (BFV) scheme, which we utilized in our framework. To better understand its functioning, equations from Equations 1–7 illustrate a simplified example of how achieving the same result is possible even after a transformation. In this example, the structure of the functions has been designed to only preserve addition, but in FHE, the same logic applies to multiplications.

Let's consider the Equation 1 and apply a homomorphic transformation (encryption) as depicted in Equation 2. To verify if the transformation occurred homomorphically, we choose two random values for *x* and *y*, as represented in Equation 3. Adding our values to Equation 1, we obtain Equation 4, from which, by performing the calculations, we arrive at Equation 5. At this point, we introduce the transformation from Equation 2, as mentioned earlier, resulting in Equation 6. By performing the last simple calculation, we can observe in Equation 7 that the result is equal on both sides, despite the transformation in between. Hence, we can conclude that this transformation was homomorphic concerning additions.

FHE applies the same logic to encryption with more complex calculations, making both additions and multiplications homomorphic. Unfortunately, in the case of non-linear calculations, FHE is not supported. Data must be decrypted before proceeding with the computation; otherwise, there is a risk of obtaining completely incorrect and unreadable results.


(1) $$\begin{array}{l} f\left(2x+3y\right)=f\left(2x\right)+f\left(3y\right) \end{array}$$

(2) $$\begin{array}{l} f\left(z\right)=6z \end{array}$$

(3) $$\{\begin{array}{l} x=-2\\ y=+4 \end{array}$$

(4) $$\begin{array}{l} f\left(2\cdot \left(-2\right)+3\cdot \left(+4\right)\right)=f\left(2\cdot \left(-2\right)\right)+f\left(3\cdot \left(+4\right)\right) \end{array}$$

(5) $$\begin{array}{l} f\left(+8\right)=f\left(-4\right)+f\left(+12\right) \end{array}$$

(6) $$\begin{array}{l} \left[6\cdot \left(+8\right)\right]=\left[6\cdot \left(-4\right)\right]+\left[6\cdot \left(+12\right)\right] \end{array}$$

(7) $$\begin{array}{l} +48=+48 \end{array}$$

### 3.2 Pyfhel library

Pyfhel (Ibarrondo and Viand, 2021) is a Python (Raschka et al., 2020) library that allows encryption using various schemes and a wide range of data while maintaining limited computational capabilities based on the chosen data type. It supports the BFV scheme and implementation on neural networks. Unfortunately, it was not designed exclusively for the field of ML. Despite being usable for neural networks, it has not been optimized for this purpose and only leverages the CPU, not utilizing the hardware acceleration possible with the GPU. Considering that encryption is already inefficient and computationally intensive, the inability to use the GPU on large datasets, such as those in Artificial Intelligence (AI), inevitably leads to very long computing processes.

#### 3.2.1 HE parameters

The implementation of the BFV scheme in Pyfhel relies on three key elements:

- *m*- represents the degree of the polynomial modulus, impacting computational capabilities and the security level of the encryption system.
- *t*- denotes the plaintext modulus, determining the size and precision of the ciphertext values for the plaintext.
- *q*- represents the ciphertext modulus, influencing the size of the ciphertext values and affecting the security and computational performance of the encryption scheme.

Balancing security and computational efficiency in FHE operations becomes possible by selecting appropriate values for these parameters. Pyfhel provides an easy-to-use interface for working with the BFV scheme, enabling encryption, computation, and decryption of data with concise and comprehensible code.

Another crucial element to consider is the Noise Budget (NB), which denotes the maximum amount of disturbance or error that can be introduced during the encryption and computation process without compromising the accuracy of the results. In operations performed on encrypted data, activities such as addition and multiplication can accumulate disturbance, putting at risk the accuracy of the results when decrypted. The NB sets a limit on how much disturbance can be tolerated before the decrypted results become unreliable. It is imperative to carefully manage and continuously monitor the NB throughout the entire computation process to ensure the security and integrity of cryptographic operations.

## 4 Datasets

The MNIST (Deng, 2012), FashionMNIST (Xiao et al., 2017), and CIFAR10 (Krizhevsky et al., 2015) datasets are popular datasets used in the ML community for training and evaluating algorithms in computer vision. Table 1 shows the main characteristics of each dataset.

**Table 1:** Summary of the main characteristics of the 3 datasets used.

**Datasets**	**MNIST**	**FashionMNIST**	**CIFAR10**
Total images	70,000	70,000	60,000
Train-set	60,000	60,000	50,000
Test-set	10,000	10,000	10,000
N° classes	10	10	10
Dimensions	28 × 28	28 × 28	32 × 32
Colors	1 - Grayscale	1 - Grayscale	3 - RGB
Classes type	Number 0-9	Clothes	Objects

### 4.1 MNIST

MNIST (Deng, 2012) is one of the most widely used datasets in ML. It consists of grayscale images of handwritten digits from 0 to 9. It represents a standard among ML datasets and is often used for basic testing. Accuracy on this dataset can easily reach high values close to 100%. In the Supplementary material, there are examples extracted from the dataset representing all 10 classes. The images appear pixelated as they are in a very small format, namely 28x28 pixels.

### 4.2 FashionMNIST

FashionMNIST (Xiao et al., 2017) is a dataset containing images of clothing items. It was created as a more complex alternative to the MNIST dataset, as it maintains the same structure but instead of handwritten digits, it features grayscale images of clothing items. Similarly, the dataset contains 70,000 images, divided into 60,000 for the training set and 10,000 for the test set, with a size of 28x28 pixels as seen in the examples in the Supplementary material.

### 4.3 CIFAR10

CIFAR10 (Krizhevsky et al., 2015) is an RGB color image dataset with dimensions of 32x32 pixels, which are slightly larger than those in the MNIST group, and consists of 10 classes of common objects and animals (see the Supplementary material). Among the datasets we used, this is the most complex, and indeed, the accuracy of various models on this dataset generally falls well below 90%. In terms of total size, it is slightly smaller than MNIST, with 50,000 images for the training set, 10,000 for the test set, totaling 60,000 data points.

## 5 PyTorch

PyTorch (Paszke et al., 2019) is an open-source library for ML developed by Facebook. It is designed to provide a flexible and scalable platform for developing AI models and is fully compatible with the Python (Raschka et al., 2020) programming language.

One of PyTorch's key features is its support for automatic gradient computation, which significantly simplifies the implementation of algorithms by allowing users to modify the network structure during program execution.

PyTorch offers various tools such as data loading and preprocessing, neural network creation, GPU training support, and integration with third-party libraries, such as Norse, which allows the creation of SNNs.

The syntax of PyTorch is clear and intuitive, making it a popular choice among ML developers. PyTorch is widely used in both academic and industrial settings for various applications, including image classification, natural language processing, computer vision, and more. Given its widespread adoption, PyTorch is continuously growing and evolving.

## 6 LeNet5 model

LeNet5 is a Convolutional Neural Network (CNN) model developed by Yann LeCun and his team at Bell Labs in the 1990s (LeCun et al., 1998). It was one of the first CNN models to be widely used for image classification and played a crucial role in the early advances of deep learning. Since then, LeNet5 has served as a foundational model for the development of more advanced CNN architectures and has found applications in various domains, including character recognition, object detection, and facial recognition.

LeNet5 is composed of convolutional, pooling, and fully connected layers. The convolutional layers extract features from the input images using convolutional filters. The pooling layers reduce the dimensionality of the extracted features while preserving their essential information. Finally, the fully connected layers classify the features and produce the output predictions. During training, the LeNet5 model utilizes error backpropagation to update the weights of the convolutional filters and fully connected layers in order to minimize the loss function (Janocha and Czarnecki, 2017) and improve the network's performance.

**Figure 4:**
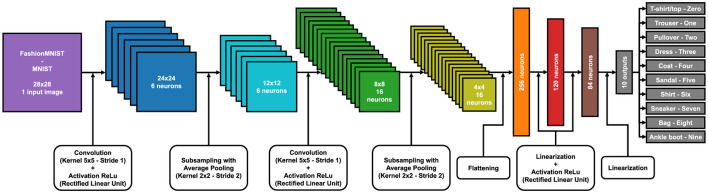
LeNet5 model with each layer and matrix size for FashionMNIST and MNIST training.

**Figure 5:**
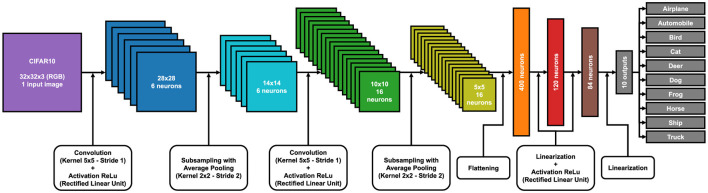
LeNet5 model with each layer and matrix size for CIFAR10 training.

In the Supplementary material, there is a 3D reconstruction of LeNet5 for the classification of the FashionMNIST and MNIST datasets. Each color represents the various layers of the model and their respective matrix dimensions, from the input image to the final output classification. In Figures 4, 5, you can see the 2D models with an explanation of the various steps for the MNIST dataset family and for CIFAR10.

### 6.1 Spiking-LeNet5 model

The Spiking-LeNet5 model (Han and Roy, 2020; Lee et al., 2016, 2019; Zenke and Ganguli, 2018; Tavanaei et al., 2019) was built based on the standard LeNet5 model. We then integrated the Norse python library with the PyTorch library to obtain the spiking version. The LeNet5, which processed each dataset differently, was modified by replacing the Rectified Linear Unit (ReLu) activation commands with the LIF activation from the Norse library, and the entire model was then placed in a timed sequence controlled by *seq*_*length*_ to allow for neuron firing.

**Figure 6:**
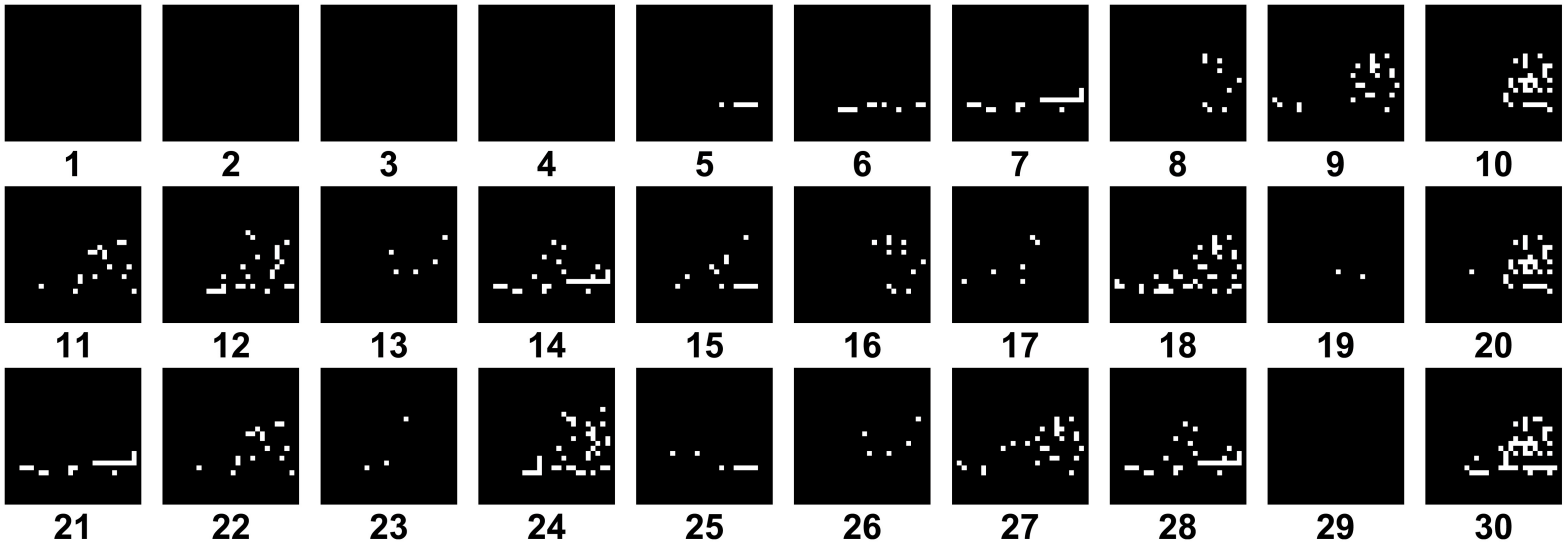
In the Spiking-LeNet5 the neurons fire randomly during the *seq*_*length*_ and the result is each time a portion of the total image, in this case it is the Ankle Boot, label 9 in the FashionMNIST dataset.

**Figure 7:**
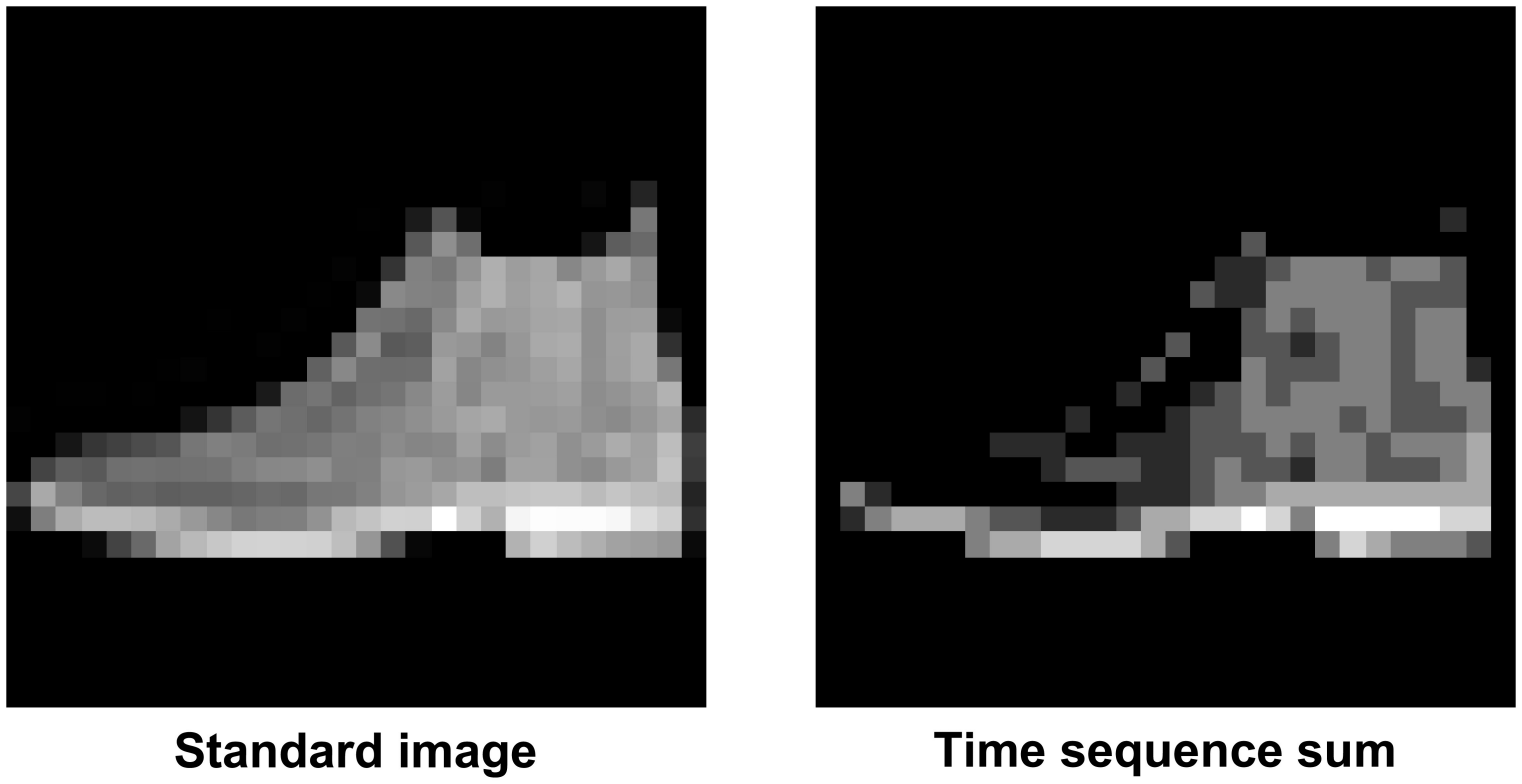
On the left we have the native Ankle Boot (Label 9 in the FashionMNIST dataset) image, while on the right there is the sum of the temporal sequence *seq*_*length*_ of Figure 6.

In Figure 6, you can see how an image from the dataset appears during the spiking temporal sequence with *seq*_*length*_ set to 30, in this case it is the Ankle Boot, label 9 in the FashionMNIST dataset. You can observe how the image only appears in certain parts because only some neurons fire at a time. In Figure 7, there is a comparison between the original image and the sum of the previous timed images. The final result is not identical, but it can be noted that during the temporal sequence, more or less all neurons fire, allowing the image to still be recognized.

## 7 Training phase

**Figure 8:**
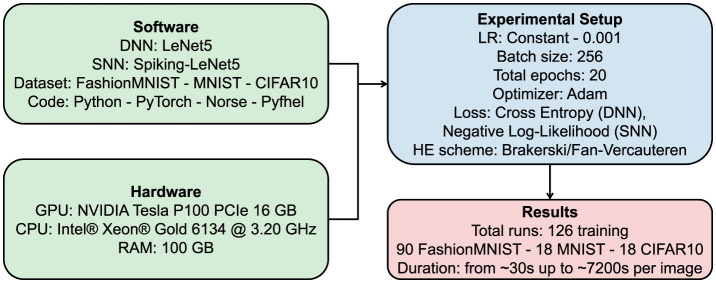
SpyKing experimental setup.

For the training phase, we set the parameters optimally to increase accuracy. The PyTorch library was chosen for defining the model, as the Norse library relies on PyTorch, allowing us to create both the LeNet5 and the Spiking-LeNet5 models based on PyTorch. The selected parameters can be seen in Table 2, and Figure 8 provides a summary diagram of the experimental setup for the SpyKing project. The learning rate was chosen using the learning rate finder technique, while the number of epochs was selected using early stopping to prevent overfitting.

**Table 2:** Training phase parameters and LIF values selected after several tests to achieve the best configuration.

**Parameters**	**LeNet5**	**Spiking-LeNet5**
Learning rate	0.001	0.001
Epochs	20	20
Batch size	256	256
Optimizer	Adam (Kingma and Ba, 2015)	Adam (Kingma and Ba, 2015)
Loss	Cross Entropy (Mao et al., 2023)	Negative Log-Likelihood (Zhu et al., 2018)
*seq*_*length*_	-	30
$\tau_{syn}^{-1}$	-	200
$\tau_{mem}^{-1}$	-	100
*v*_*leak*_	-	0
*v*_*th*_	-	0.5
*v*_*reset*_	-	0
Encoder	-	Constant Current LIF

The LIF parameters are similar to the default ones provided by Norse.

**Figure 9:**
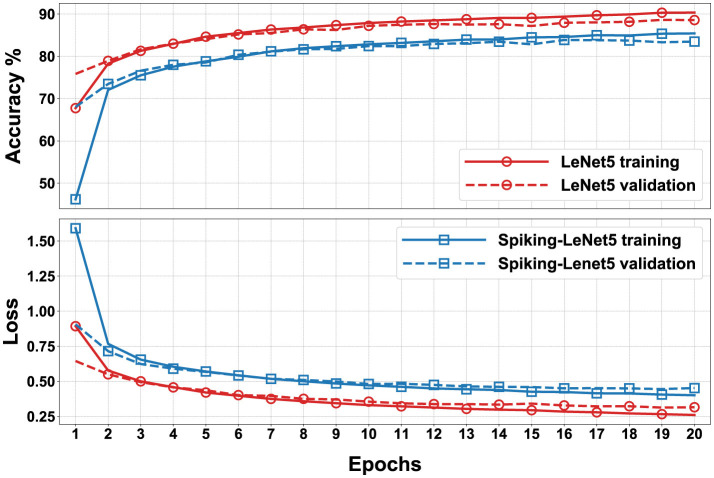
Accuracy and loss during training and validation of LeNet5 and Spiking-LeNet5 for the FashionMNIST dataset. The figure shows accuracy and loss values across different training epochs.

In Figure 9, we can observe the accuracy and loss (Janocha and Czarnecki, 2017) for each epoch during the training on the FashionMNIST dataset, comparing LeNet5 and Spiking-LeNet5 (Meftah et al., 2013). Additionally, the dashed lines illustrate how, for each model, validation has slightly lower performance compared to training. Furthermore, we can notice that the spiking model has slightly lower final accuracy compared to the non-spiking model, which is due to the intrinsic complexity of the spiking version. Also, the computation time of the spiking model differs from that of LeNet5; on average, the spiking model takes the same time as LeNet5 multiplied by the value of *seq*_*length*_. The respective training graphs for the MNIST and CIFAR10 datasets are visible in Supplementary material.

As can be observed, the final accuracy achieved by the standard LeNet5 model varies across the datasets: it's around ≈99% for MNIST, ≈80-90% for FashionMNIST, and ≈60-70% for CIFAR10. This disparity among the datasets arises from practical reasons; MNIST, being the simplest dataset, exhibits the highest accuracy. FashionMNIST is similar to MNIST but with slightly more complex classes to distinguish. Lastly, CIFAR10 is a dataset with 3 RGB channels and consequently much more complex than the previous two, resulting in lower model accuracy on this dataset as well. Given the differences between the datasets and the repeated trials for all, to avoid overwhelming subsequent paragraphs, the following discussion will focus more on the FashionMNIST dataset, which has intermediate complexity, while the results of the other two datasets can be found in the Supplementary material.

### 7.1 Parameters selection

**Figure 10:**
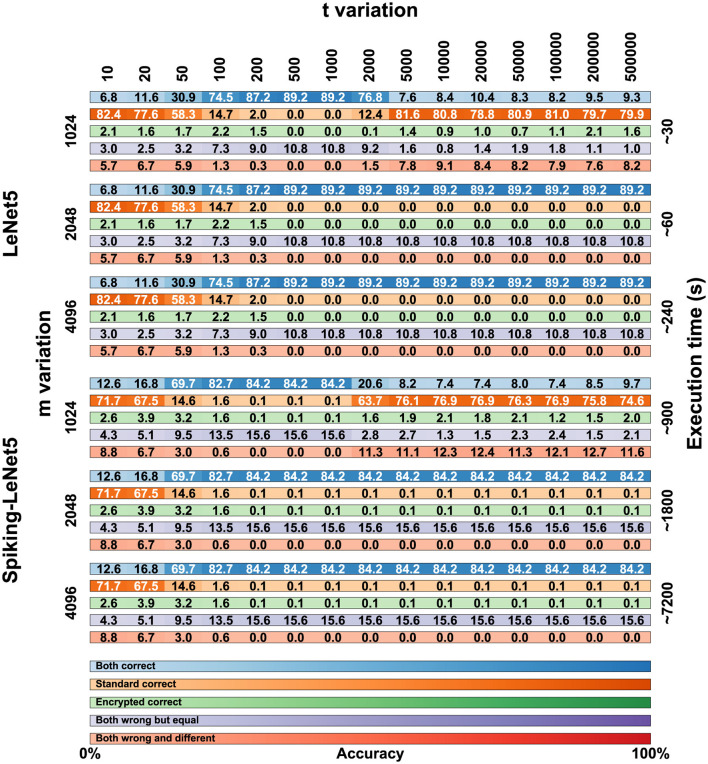
Comparison matrix for *t* and *m* variation for the FashionMNIST dataset on encrypted LeNet5 and Spiking-LeNet5 models.

After training, in order to proceed with encryption, it is necessary to define the parameters of the BFV scheme: *m*, *t*, and *q*. The parameter *m* must be a power of 2 greater than 1024 and is directly proportional to the NB. Values of *m* that are too high would lead to overly complex computational calculations, while low values would be too insecure. Values of *m* equal to 2048 or higher do not significantly alter the results but exponentially increase computation times. Therefore, we performed these calculations only on the FashionMNIST dataset, and the results are visible in Figure 10.

The value of *t* can also vary, but too low values lead to incorrect encryption, while too high values degrade the results, making them unreadable. For the FashionMNIST dataset, we evaluated a variation of *t* on 15 values between 10 and 500,000, noting that after the value of 5,000 there are no significant differences. Consequently, for the other two datasets, we evaluated the results between 10 and 5,000.

The last parameter is *q*, but it is related to *m* in determining the value of NB and is automatically calculated by the Pyfhel library to obtain adequate encryption.

The NB also allows for a certain tolerance in operations before the results degrade too much, and therefore sometimes it needs to be *recharged* by decrypting and encrypting again. However, this did not affect our results because, as we will see later, due to nonlinear calculations in the models, we were forced to decrypt and encrypt multiple times. Consequently, the value of NB was replenished each time, allowing us to perform subsequent encrypted calculations without issues.

### 7.2 Encryption

In Table 3, there are comparisons for the computation times for each dataset. As can be seen, with the hardware available to us and with a value of *m* set to 1024, it takes approximately 1 second to encrypt an image from the FashionMNIST and MNIST datasets for the LeNet5 model, and about 30 seconds for the Spiking-LeNet5 model. After that, it takes another 30 seconds for evaluating the image on the encrypted LeNet5 model and about 15 minutes on the encrypted Spiking-LeNet5 model. The value of 15 minutes is obtained by multiplying the 30 seconds taken by LeNet5 by the value of *seq*_*length*_, which in our case is 30. It can also be noted that increasing the value of *m* results in an exponential increase in computation time, while the variation in the parameter *t* has no significant effect.

**Table 3:** Encryption and execution time for each image with respect to the variation of the model and the *m* parameter from 1,024 to 4,096.

**Datasets**	**Time (seconds)**	**LeNet5**			**Spiking-LeNet5**		
		**1,024**	**2,048**	**4,096**	**1,024**	**2,048**	**4,096**
Fashion MNIST	Encryption	1	2	8	30	60	240
	Plaintext execution	0.03	0.03	0.03	1	1	1
	Encrypted execution	30	60	240	900	1,800	7,200
MNIST	Encryption	1	2	8	30	60	240
	Plaintext execution	0.03	0.03	0.03	1	1	1
	Encrypted execution	30	60	240	900	1,800	7,200
CIFAR10	Encryption	2	4	16	60	120	480
	Plaintext execution	0.07	0.07	0.07	2	2	2
	Encrypted execution	60	120	480	1,800	3,600	14,400

In Table 4, there is an estimation of the execution time, based on the same hardware, for other types of models, considering only the FashionMNIST dataset. As can be seen, the time is proportional to the number of parameters handled by the model itself, and even with models slightly more complex than LeNet5, much longer computation times are obtained.

**Table 4:** Prediction time for each image of the FashionMNIST dataset reported in seconds for each model with *m* = 1,024.

	**Time (seconds)**	**LeNet5**	**AlexNet**	**VGG16**	**ResNet50**
Complexity		60 k	60 M	138 M	23 M
Standard	Encryption	1	60	140	20
	Plaintext execution	0.03	30	70	10
	Encrypted execution	30	30 k	70 k	10 k
Spiking	Encryption	30	1.8 k	4.2 k	600
	Plaintext execution	1	1 k	2.1 k	300
	Encrypted execution	900	900 k	2.1 M	300 k

The long processing time of encrypted data are due to the complexity of the encrypted computations and it also depends on the complexity of each model (N° of parameters).

### 7.3 Resources

The hardware resources available for conducting the experiments consisted of a NVIDIA Tesla P100 PCIe 16 GB GPU, an Intel^®^ Xeon^®^ Gold 6134 @ 3.20 GHz CPU, and 100 GB of RAM.

The code (available at this GitHub address: https://github.com/farzadnikfam/SpyKing) was entirely written in Python with the help of various libraries, including PyTorch, Norse, and Pyfhel.

## 8 Results

In Figure 10, all the numerical data in percentage of the results obtained on the FashionMNIST dataset are presented in the form of a matrix. The simulations were conducted on 15 variations of *t* ranging from 10 to 500,000 and with 3 variations of *m*: 1,024, 2,048, and 4,096. The calculations were performed for both LeNet5 and Spiking-LeNet5 models and were divided based on accuracy between plaintext and encrypted models. Since, as can be seen, the results with *m* set to 4,096 are identical to those with *m* set to 2,048, for both the standard and spiking models, the case with *m* set to 4096 will not be considered from now on.

**Figure 11:**
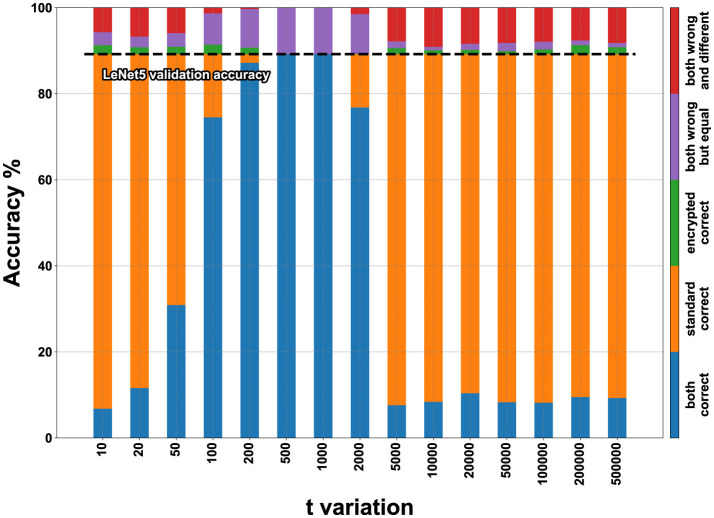
FashionMNIST accuracy on encrypted LeNet5 for *t* variation with *m* set to 1,024.

**Figure 12:**
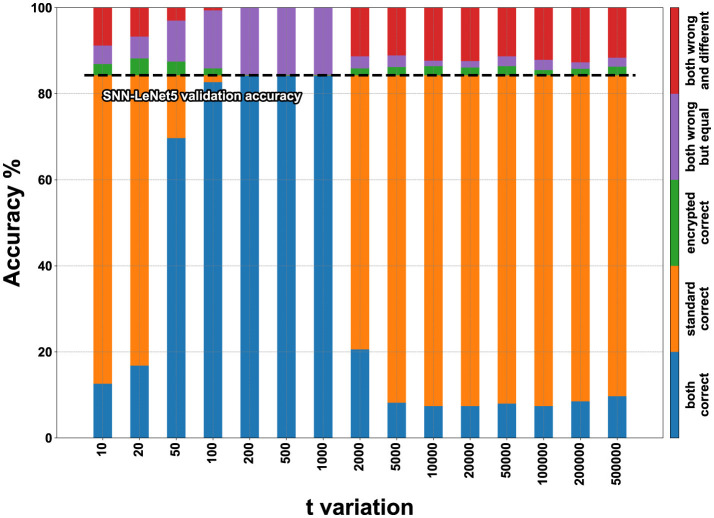
FashionMNIST accuracy on encrypted Spiking-LeNet5 for *t* variation with *m* set to 1,024.

In Figures 11, 12, the visual representation of the same matrices can be seen with bar graphs to better understand the results. The results in matrix form for the FashionMNIST (with *m* = 2,048), MNIST and CIFAR10 datasets are in Supplementary material, and the respective bar graphs have been grouped with those of FashionMNIST (with *m* = 1,024) in Supplementary material for better comparison.

To better understand how to read the matrices and bar graphs, here is an explanation of the colors:

- Blue (both correct)–represents the percentage of images classified correctly by both the plaintext and encrypted models.
- Orange (standard correct)–indicates the percentage of data classified correctly by the plaintext model but not by the encrypted one. It can be noticed that by adding the percentages of blue and orange colors, the same accuracy value is always obtained, whether changing *m* or changing *t*. This data represents the accuracy value of validation during training, which in the case of FashionMNIST corresponds to 89.2% for LeNet5 and 84.3% for Spiking-LeNet5.
- Green (encrypted correct)–this percentage is the inverse counterpart of the previous one, meaning the images were classified correctly by the encrypted model but not by the plaintext model. The percentages are generally low and almost insignificant, as this occurs because the encrypted model classifies differently from the plaintext model, which is incorrect, but by pure coincidence chooses the correct label. Therefore, this small percentage has no statistical value but is merely coincidental, as the encrypted model should classify like the plaintext model, even if the latter is wrong.
- Purple (both wrong but equal)–represents the case where the encrypted model and the plaintext model coincide but have not classified the correct label. This data is important because it shows how the encrypted model has functioned correctly by mimicking the plaintext model, even if the initial classification was incorrect.
- Red (both wrong and different)–this last situation shows the case where both the encrypted and plaintext models have made mistakes and are different from each other. So, the label has not been correctly classified by either of the two models, and furthermore, the encrypted one has not copied the plaintext one. This percentage represents the worst-case scenario where nothing has worked as it should.

## 9 Discussion

**Figure 13:**
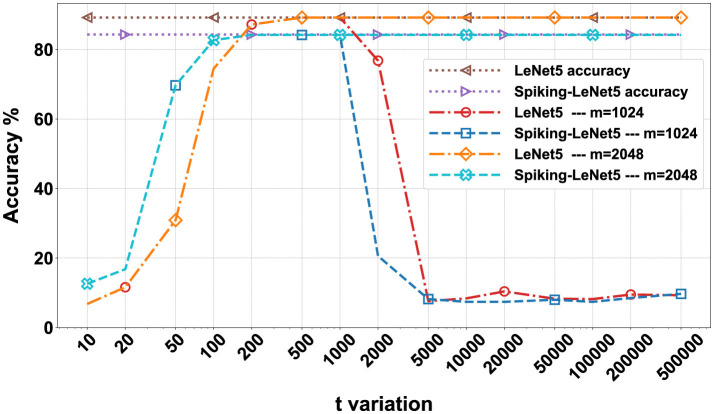
Comparison of FashionMNIST accuracy between plaintext and encrypted versions of LeNet5 and Spiking-LeNet5 for *t* variations when both plaintext and encrypted versions classified correctly.

To better discuss the results obtained in the previous section, we can refer to Figure 13, where the most relevant data has been presented in the form of graphs. Specifically, we compared, varying *t*, the accuracy of the LeNet5 and Spiking-LeNet5 models in both plaintext and encrypted versions, with the parameter *m* set to 1,024 and 2048. To simplify, we essentially graphically represented the accuracy previously marked in Blue–both correct, i.e., when the encrypted model achieved the same results as the plaintext model and both coincided with the correct labels.

**Figure 14:**
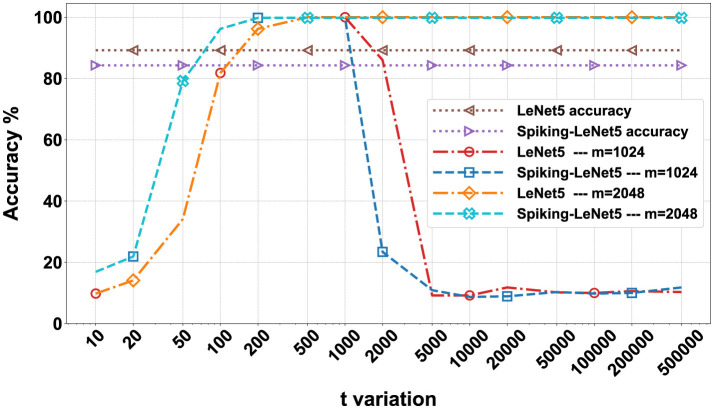
Comparison of FashionMNIST accuracy between plaintext and encrypted versions of LeNet5 and Spiking-LeNet5 for *t* variations when both plaintext and encrypted versions coincide in both correct and incorrect classification.

As we can see, both the standard and spiking versions reach approximately maximum accuracy, that is, the validation accuracy during training, with *t* values ranging from 200 to 1,000. From this value onwards, the models with *m* set to 1,024 show results that degrade quickly, while models with *m* set to or higher than 2048 maintain maximum accuracy. Apart from this difference between *m* set to 1,024 and higher values, there are no other differences in the initial part, but especially high values of *t* are not so relevant because they indicate a high level of encryption that can increase computational costs or degrade data. The most important part is the part of the graph representing the lower *t* values, those below 200, where we can see how the spiking model performs significantly better than the standard model. Of course, these results are limited by the fact that the final accuracy of the validation of the spiking model is lower than that of the LeNet5 even in the plaintext version, which is why we created the graph in Figure 14.

In Figure 14, we no longer compare only the Blue - both correct percentages, but we add these to those of Purple - both wrong but equal. In practice, we added all the cases where the encrypted model correctly provided the same result as the plaintext model, whether the latter was correct or not. In fact, the goal of this research was not only to demonstrate the feasibility of encrypted models but also their reliability, and considering that with certain combinations of *t* and *m*, values close to 100% correctness between the encrypted and plaintext models can be achieved, I would say that the result has been achieved. Specifically, we can see that in Figure 14, both the standard and spiking models in the encrypted versions reach 100% accuracy in emulating the plaintext versions, maintaining approximately the same shape as Figure 13. This means that even in this case, for low *t* values, the Spiking-LeNet5 model performs better than the LeNet5.

In conclusion, SNNs react better to encryption than DNNs, making them more secure for data encryption. However, they still have some criticalities: first of all, they have an intrinsic latency time, the *seq*_*length*_ parameter, that significantly lengthens computation times, and secondly, they generally have lower validation accuracy. The same results can also be viewed for the MNIST and CIFAR10 datasets in Supplementary material.

### 9.1 Models encryption

One of the main problems of FHE is that it can only work with linear calculations of addition and multiplication. The LeNet5 model, as we have implemented it in Figure 4, also includes non-linear calculations: ReLu activations. Currently, this part of calculations cannot be achieved with the encrypted model, so every data pass through the activation layer must be decrypted first and then re-encrypted. Obviously, these steps lead to a model that is not fully encrypted and to data vulnerability during the activation phase. In fact, this research aspect falls within future projects to improve the model.

**Figure 15:**
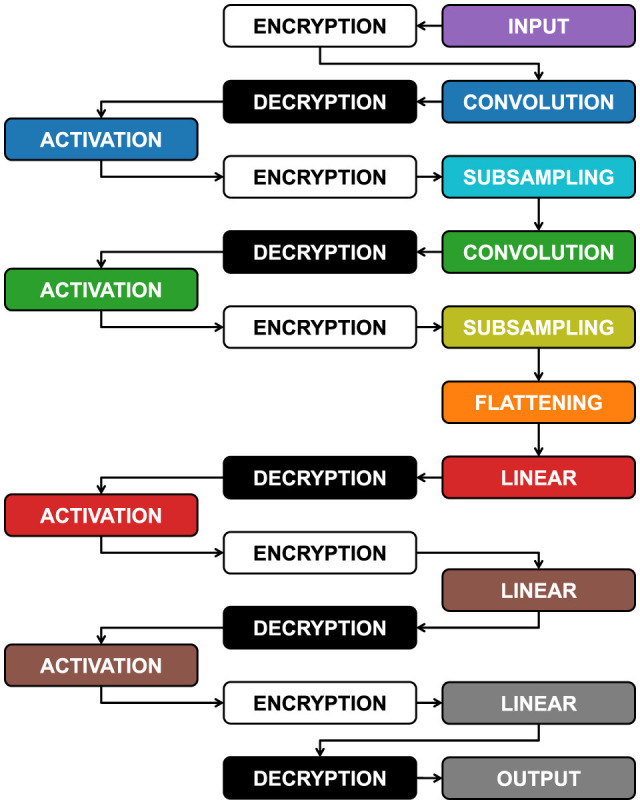
Inside the LeNet5 we need to decrypt and encrypt again four times because the activation function ReLu is not a linear calculation.

In Figure 15, we can see how the encrypted model actually behaves. The steps are the same for both the standard and the spiking model and apply to all datasets. The color codes used are the same as those used in Figure 4 for better understanding. As can be seen, the data must be decrypted and re-encrypted 4 times during the entire process, in addition to the initial encryption and final decryption. Considering that the computation times of an encrypted model are up to 1000 times slower than plaintext, both in the standard and spiking cases, one can imagine how impactful these encryption steps due to activations are.

#### 9.1.1 Noise Budget values

However, these continuous encryptions also have a positive aspect. As mentioned in the Section 3.2.1, the NB degrades every time linear calculations are performed, and if it reaches zero, the data would become unreadable. The continuous encryption during the process allows the NB to be recharged each time, enabling encrypted calculations without repercussions on the final accuracy.

**Figure 16:**
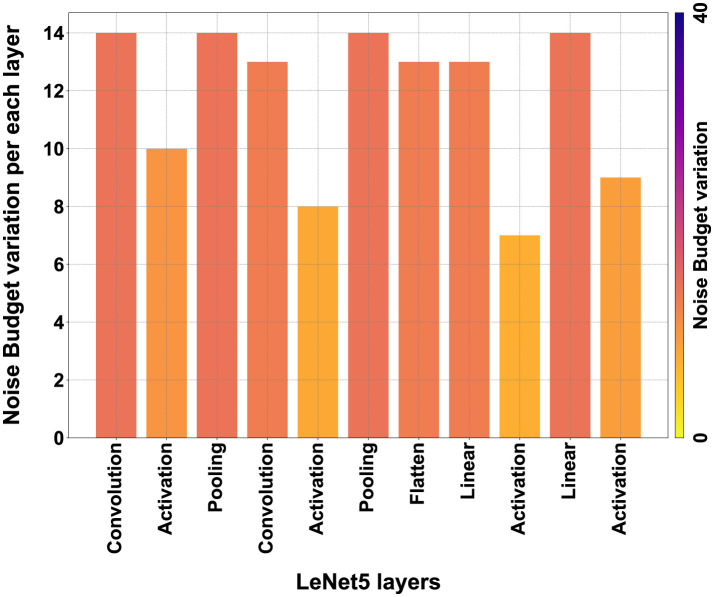
NB qualitative variation during the process across the layers.

**Figure 17:**
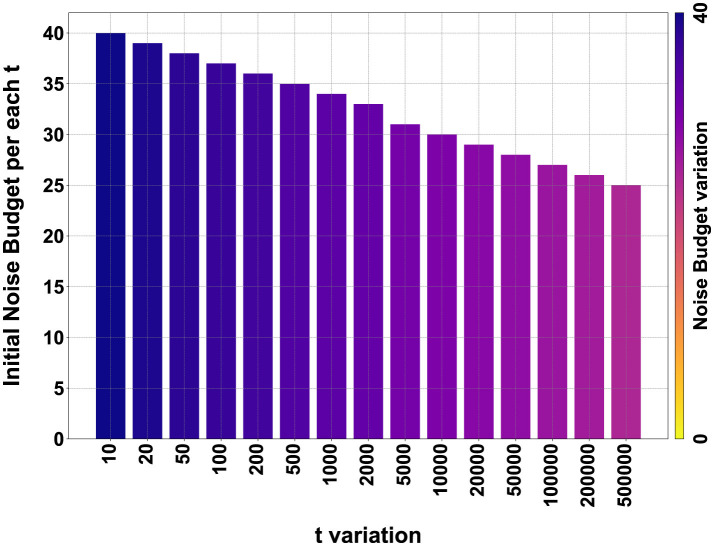
NB qualitative variation for each *t* variation.

In Figure 16, we can qualitatively see the amount of NB during the various layers, and it can be observed how it reloads after each activation due to the new encryption. In Figure 17, we can see qualitatively how the NB value is not independent of *t*, but rather, for high values of *t*, i.e., high encryption, the initial NB value is lower, and therefore fewer calculations can be absorbed, while with low values of *t*, the NB is higher with greater manipulation possibilities. Figures 16, 17 were extrapolated from the overall graph shown in Supplementary material, where all numerical data are reported, and it can be noted that NB does not depend solely on *t* but also on *m*. In fact, higher values of *m* lead to higher NB values, allowing more calculations, but at the same time, drastically increasing computation time.

### 9.2 Confusion matrices

**Figure 18:**
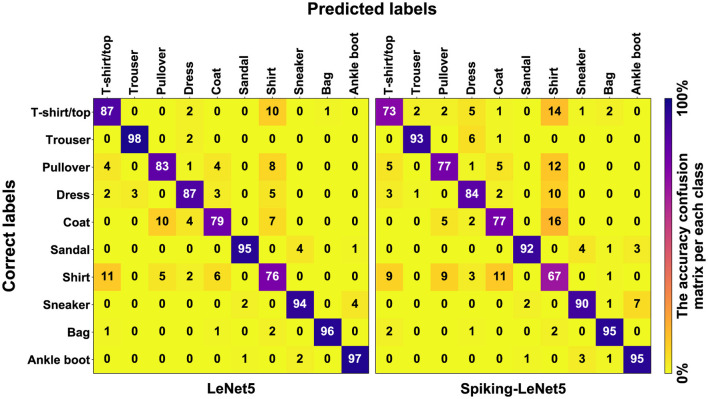
Plaintext confusion matrix for FashionMNIST. The *Shirt* class is the one that misleads the model the most.

In Figure 18, the confusion matrices of both the standard and spiking models for the FashionMNIST dataset are depicted. It can be observed that in both cases, the matrix is fairly orderly between predicted classes and correct labels. The only class that creates slight confusion for the models is class number 6, representing the *Shirt*.

**Figure 19:**
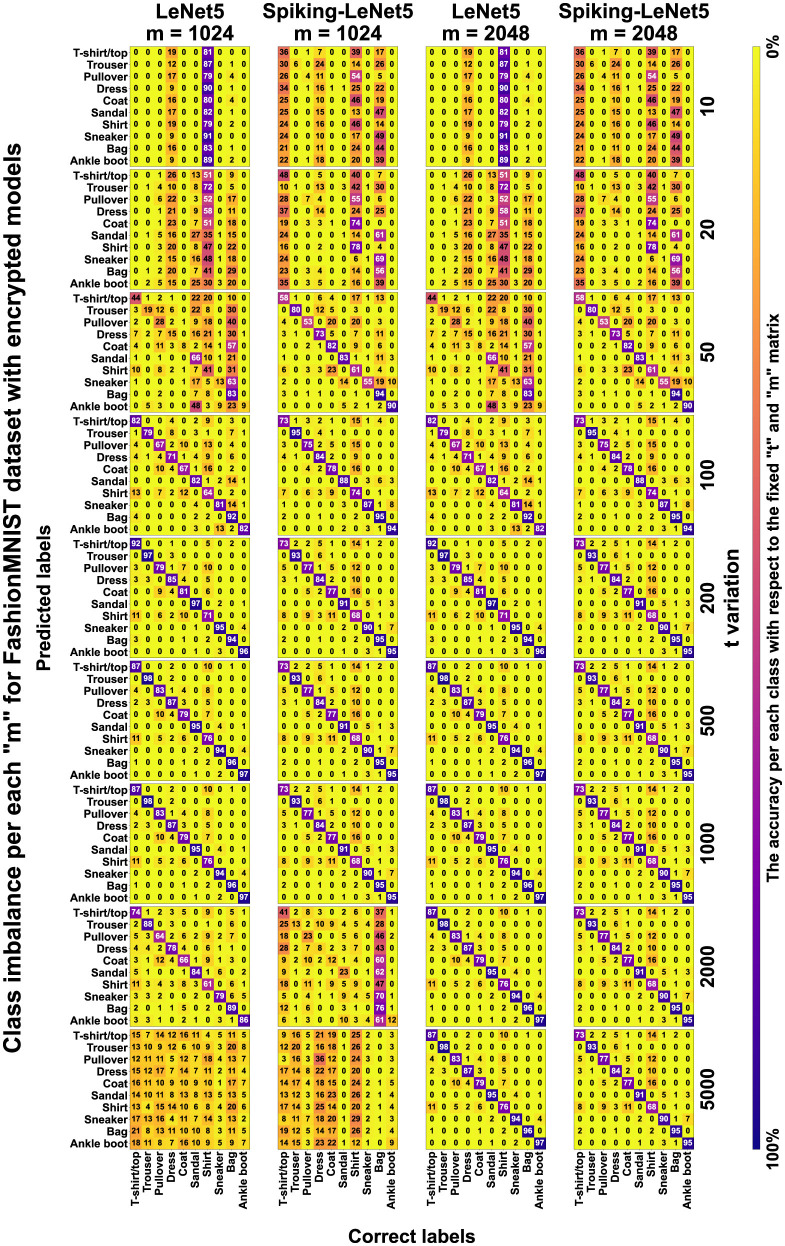
Encrypted confusion matrix for FashionMNIST with *t* and *m* variation. It can be noticed that for low values of *t*, the results tend to concentrate on labels that resemble each other the most. Spiking-LeNet5 is less random than LeNet5 for low values of *t*.

Now, looking at Figure 19, we can see all the confusion matrices of the LeNet5 and Spiking-LeNet5 models in the encrypted case, with all variations of *t* and *m*. It is easy to notice that for *m* values equal to 1,024, the results degrade quickly with values of *t* that are too high, mostly resulting in random results equivalent to overly encrypted and no longer readable data. On the other hand, with *m* values equal to 2,048 or higher, the results remain constant and unchanged, but obviously the excessive complexity of encryption makes calculations slower and more difficult. Instead, for low *t* values, the results are confusing but less random and tend to accumulate on certain classes, especially on the *Shirt* class, as in the plaintext case. Moreover, it can be noticed that they perform better in the spiking version since the matrices stabilize for lower values of *t*. Obviously, the classes on which the results accumulate depend on the shape and object represented by the class itself. In the case of FashionMNIST, it can be easily inferred that the *Shirt* class is the most confusing for the model, as it can be easily assimilated to other classes.

In Supplementary material, the plaintext and encrypted confusion matrices for the MNIST and CIFAR10 datasets are displayed. In this case, it is evident how for MNIST, the most confusing class is class 8, which is visually more complex than all the other numbers and therefore more easily misleads the models, being able to resemble any other number. It should also be noted that all these confusion matrices reflect the graphs shown in Figures 10–14, showing the correspondence of various accuracies and the different behavior for different values of *m* and *t*.

### 9.3 Layer errors

**Figure 20:**
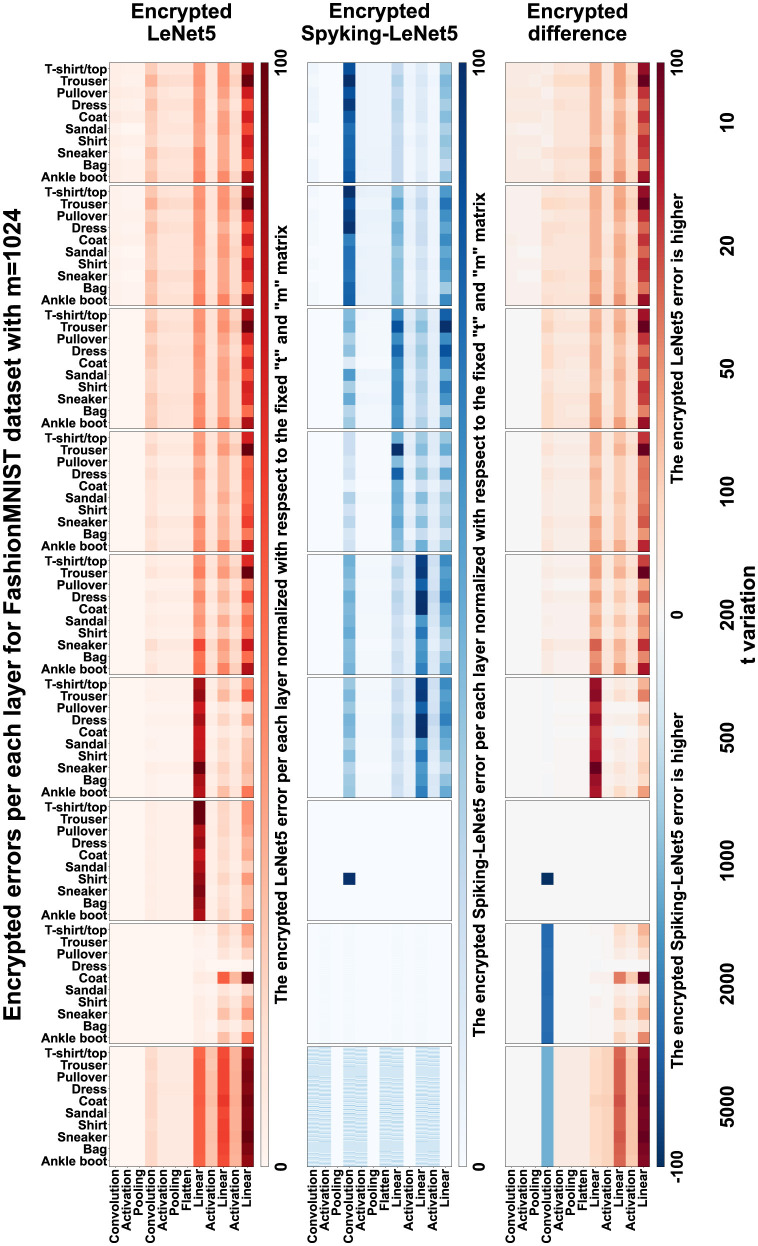
Errors layer-by-layer with FashionMNIST and *m* = 1,024. The top Red strip represents the errors in the layers of the LeNet5, the Blue strip in the middle represents the errors in the layers of the Spiking-LeNet5. The last strip at the bottom represents the difference between the errors in the layers of LeNet-5 and Spiking-LeNet5. It can be noticed that the third strip is predominantly Red, indicating that Spiking-LeNet5 generally performs better.

In the matrices of Figure 20, the normalized layer-by-layer errors are represented. Normalization was performed with values ranging from 0 to 1 for each individual matrix. Naturally, for low and high values of *t*, errors are much higher, even in the order of tens of times, compared to central *t* values, but normalizing each matrix separately served to show the differences between individual layers and especially between LeNet5 and Spiking-LeNet5. This way, it is better appreciated which model performs better and which layers accumulate more errors. Total normalization across all *t* values would not have allowed to notice the differences, given the huge difference between the central *t* values and the most extreme ones.

Observing the matrices, we can notice that errors mainly accumulate in the final layers, especially in the linearization layers, precisely because there are more calculations for reducing large matrices to the final linear array of 10 classes. It can also be noted that there are not many errors in the activation layers because during activations, the models are decrypted and there is a lower accumulation of errors. Furthermore, it is noted that there are no significant differences between the various classes and more or less all have the same error values in the various layers, with a greater accumulation in the final linearizations.

To better understand Figure 20, here is an explanation of the strips:

- in the first strip (the Red one) there are the errors produced by the encrypted LeNet5.
- in the second strip (the Blue one) there are the errors produced by the encrypted Spiking-LeNet5.
- in the third strip, the difference between the errors of the standard model and the spiking model was calculated, the normalization in this case was performed after the calculation of the difference. The Red parts show that there was a greater error in LeNet5, vice versa the Blue parts show that Spiking-LeNet5 made more mistakes.

Looking at the third strip of Figure 20, it can be noticed that it is mainly Red, which means that generally Spiking-LeNet5 performed better.

The sporadic squares much denser than those of other classes or layers generally show those classes that mislead the models the most under certain conditions. For FashionMNIST (see Figure 20), for example, a dense square can be seen in the *Shirt* class with *m* equal to 1,024 and *t* equal to 1,000.

## 10 Conclusion

In this work, we aimed to provide a comparison between classical models like DNNs and the less commonly used and less known SNNs, additionally leveraging FHE to assess their effectiveness and practicality in realistic scenarios. The final outcome demonstrated how, under certain conditions, SNNs are indeed more efficient than DNNs, and how FHE can enable the manipulation of sensitive data without the risk of intrusions. However, this work needs to be further developed to address some of its most glaring limitations:

- the inability to use encrypted data in the nonlinear phases of a model (Marchisio et al., 2020).
- the slowness attributed to encryption, particularly pronounced in this specific case since the Pyfhel library operates solely on CPU.
- the latency of SNNs, which precludes the application of these studies to real-time cases.

Therefore, the next steps in this field involve developing accelerated encryption models using GPUs and conducting in-depth research to overcome the issue of nonlinear computations. Furthermore, SNNs are still in their infancy, and undoubtedly, there will be more opportunities for their utilization in the future, especially when latency becomes less of a factor due to advancements in computing power.

## Data Availability

The original contributions presented in the study are included in the article/Supplementary material, further inquiries can be directed to the corresponding author.
