# Supplementary material for: SpyKing—Privacy-preserving framework for Spiking Neural Networks
Source: Front Neurosci. 2025 May 30;19:1551143. doi: 10.3389/fnins.2025.1551143 (PMC12162590; doi:10.3389/fnins.2025.1551143)
Supplement: Supplementary file 1 [file Data_Sheet_1.pdf]

Supplementary Material

1 SUPPLEMENTARY TABLES AND FIGURES

In this supplementary material, all the results related to the SpyKing research have been provided, which were not included in the main paper to avoid overburdening the argumentation. Since the SpyKing paper mainly contained results for the FashionMNIST dataset, this supplementary material presents the figures and graphs related to the MNIST and CIFAR10 datasets. The following images are simply an ordered list corresponding to SpyKing paper, along with their descriptions. For a complete explanation, refer to the text in SpyKing paper, which also includes references to the images in this supplementary material.

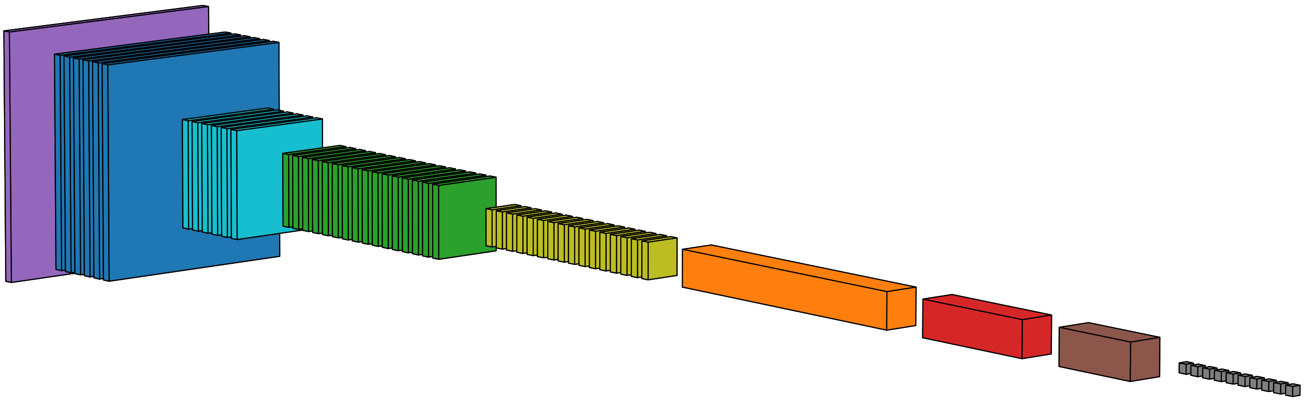

Figure S1: LeNet5 3D-model for the FashionMNIST and MNIST datasets. Each color represents a layer and the squares are the matrices dimensions during the training. For a better explanation see the 2D-models explained in the SpyKing paper.

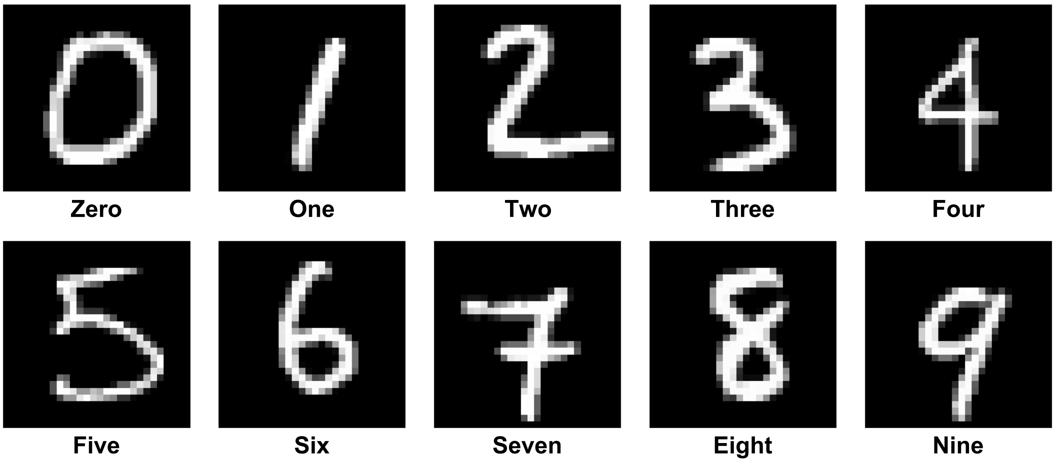

Figure S2: An example for each class of the MNIST dataset.

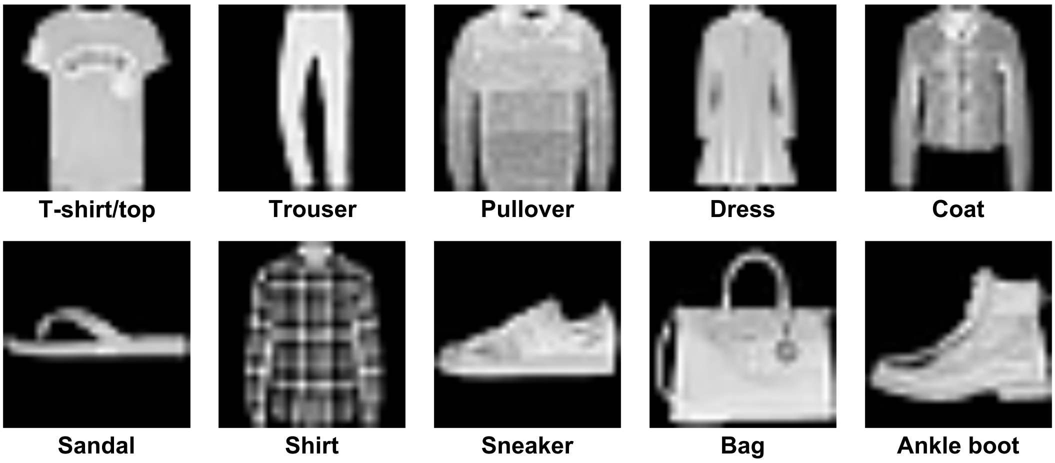

Figure S3: An example for each class of the FashionMNIST dataset.

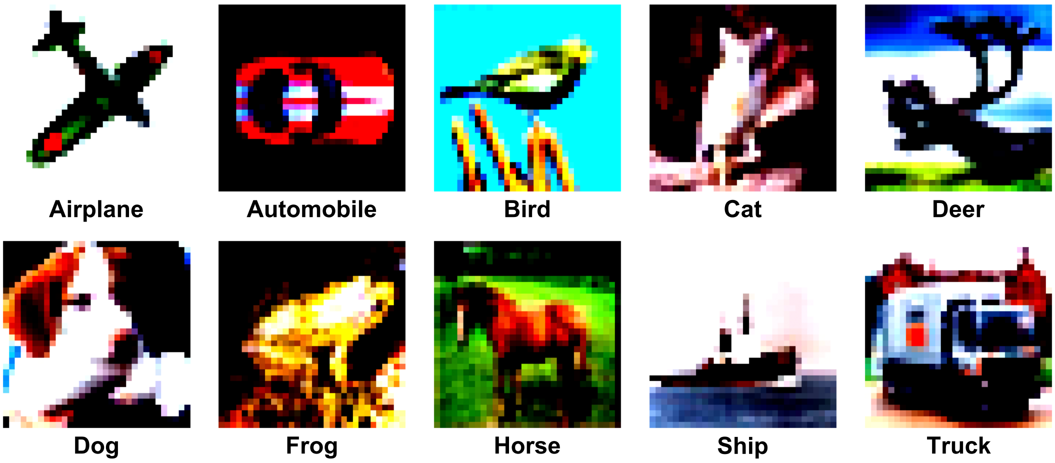

Figure S4: An example for each class of the CIFAR10 dataset.

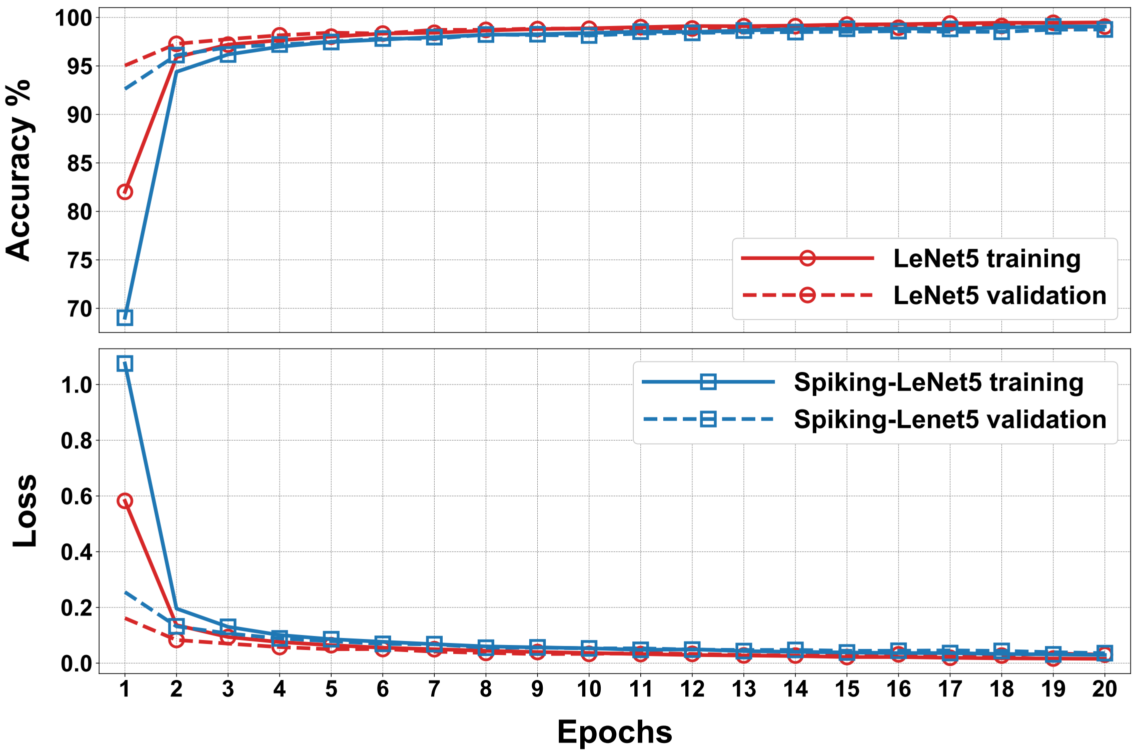

Figure S5: Accuracy and loss during training and validation of LeNet5 and Spiking-LeNet5 for the MNIST dataset. The figure shows accuracy and loss values across different training epochs.

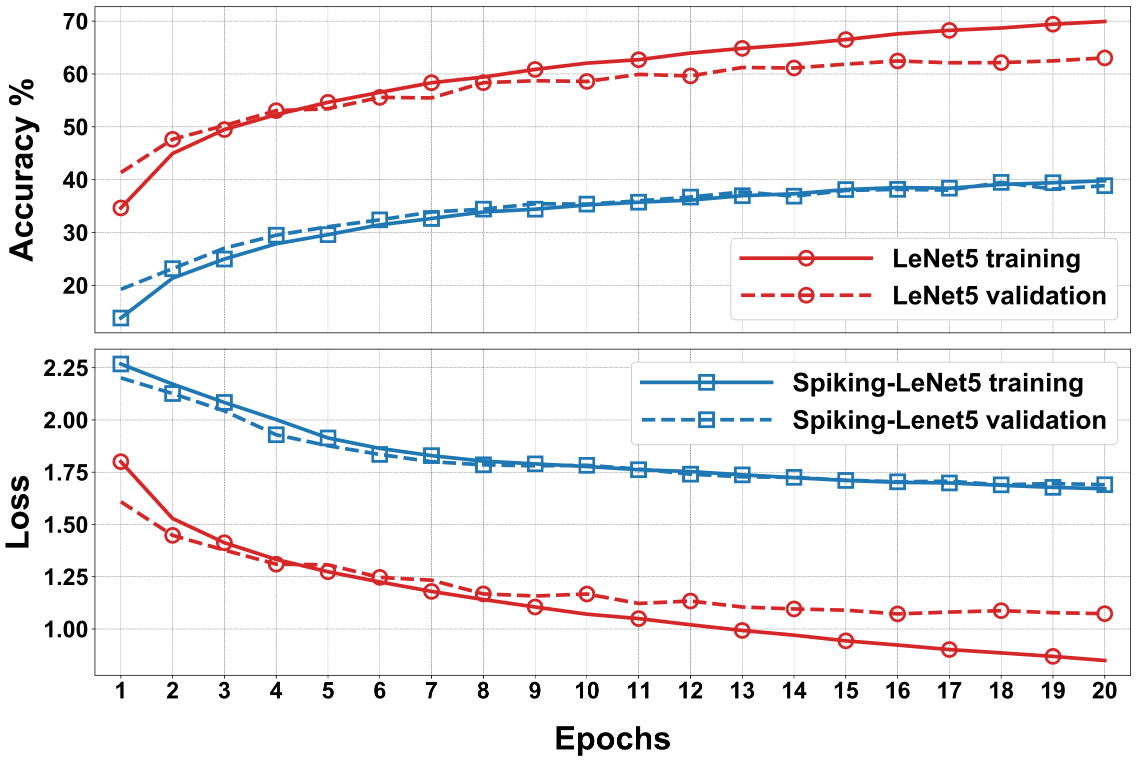

Figure S6: Accuracy and loss during training and validation of LeNet5 and Spiking-LeNet5 for the CIFAR10 dataset. The figure shows accuracy and loss values across different training epochs.

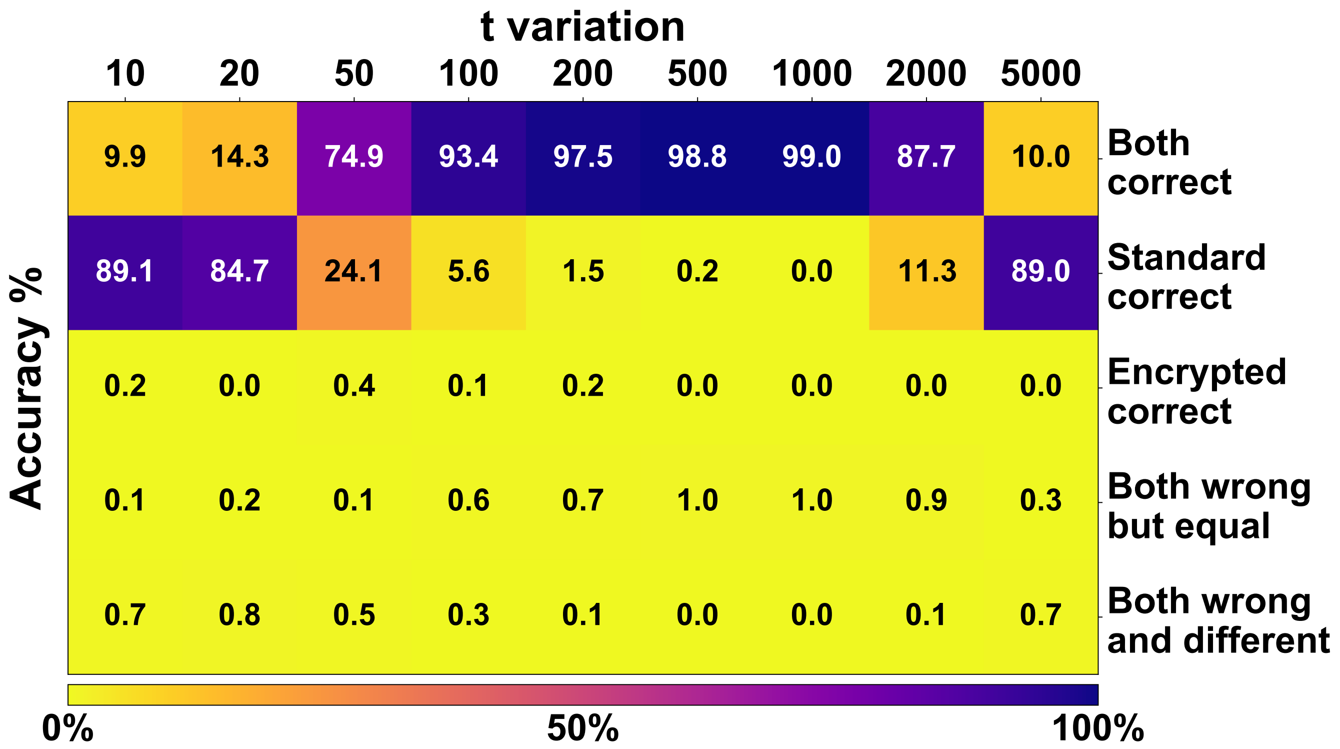

Figure S7: Comparison matrix for t variation and m set to 1024 for the MNIST dataset on encrypted LeNet5 model.

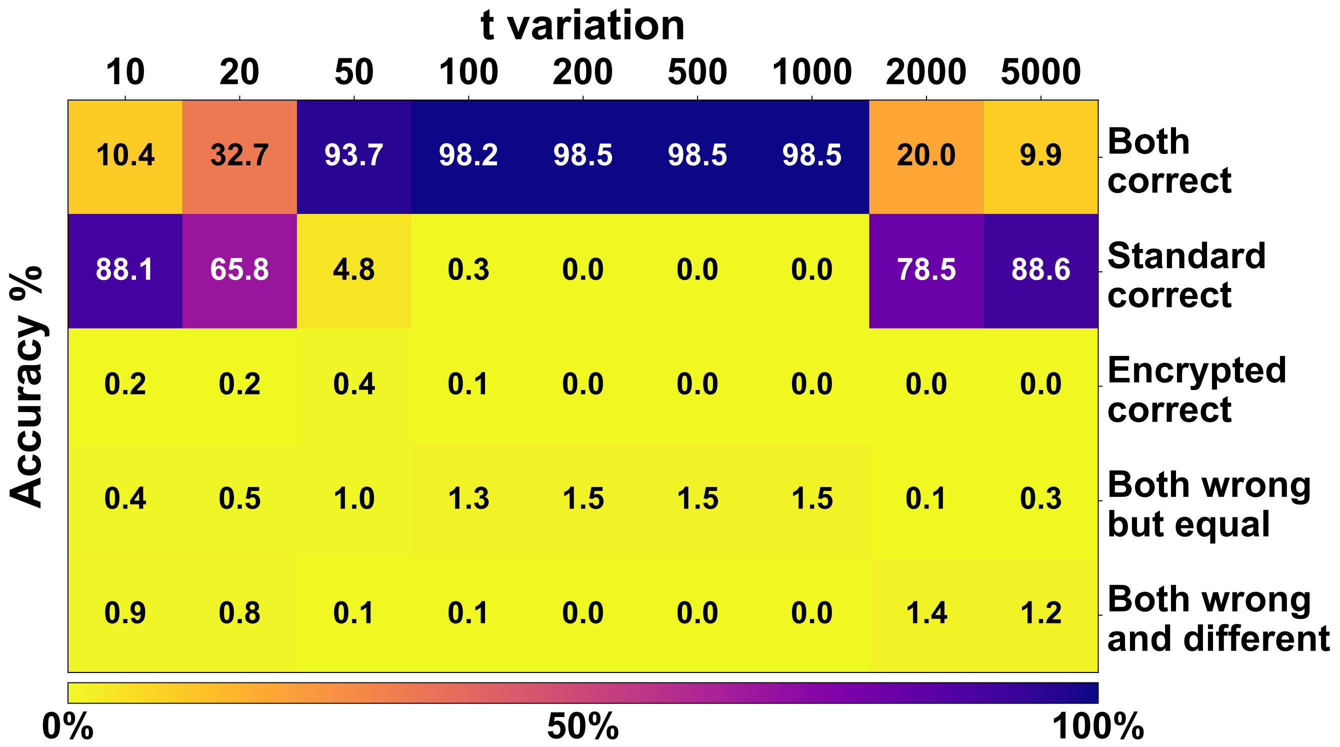

Figure S8: Comparison matrix for t variation and m set to 1024 for the MNIST dataset on encrypted Spiking-LeNet5 model.

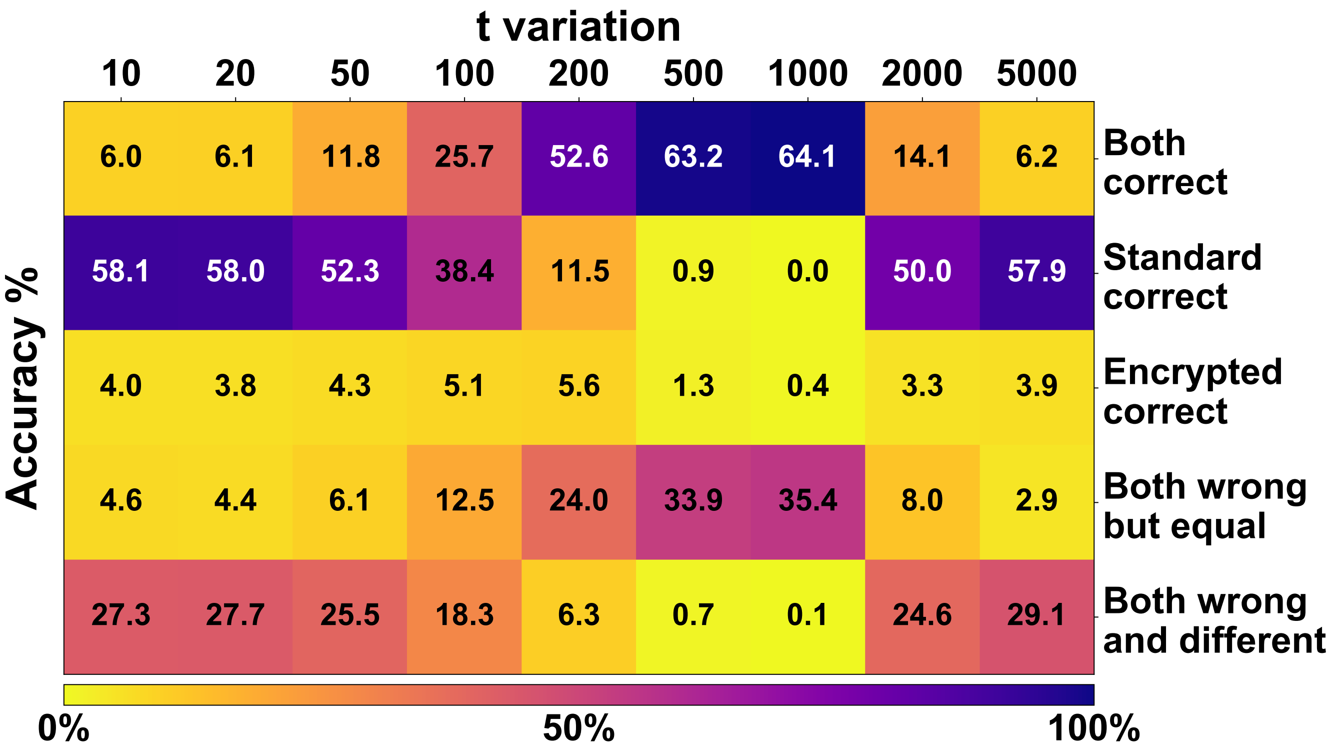

Figure S9: Comparison matrix for t variation and m set to 1024 for the CIFAR10 dataset on encrypted LeNet5 model.

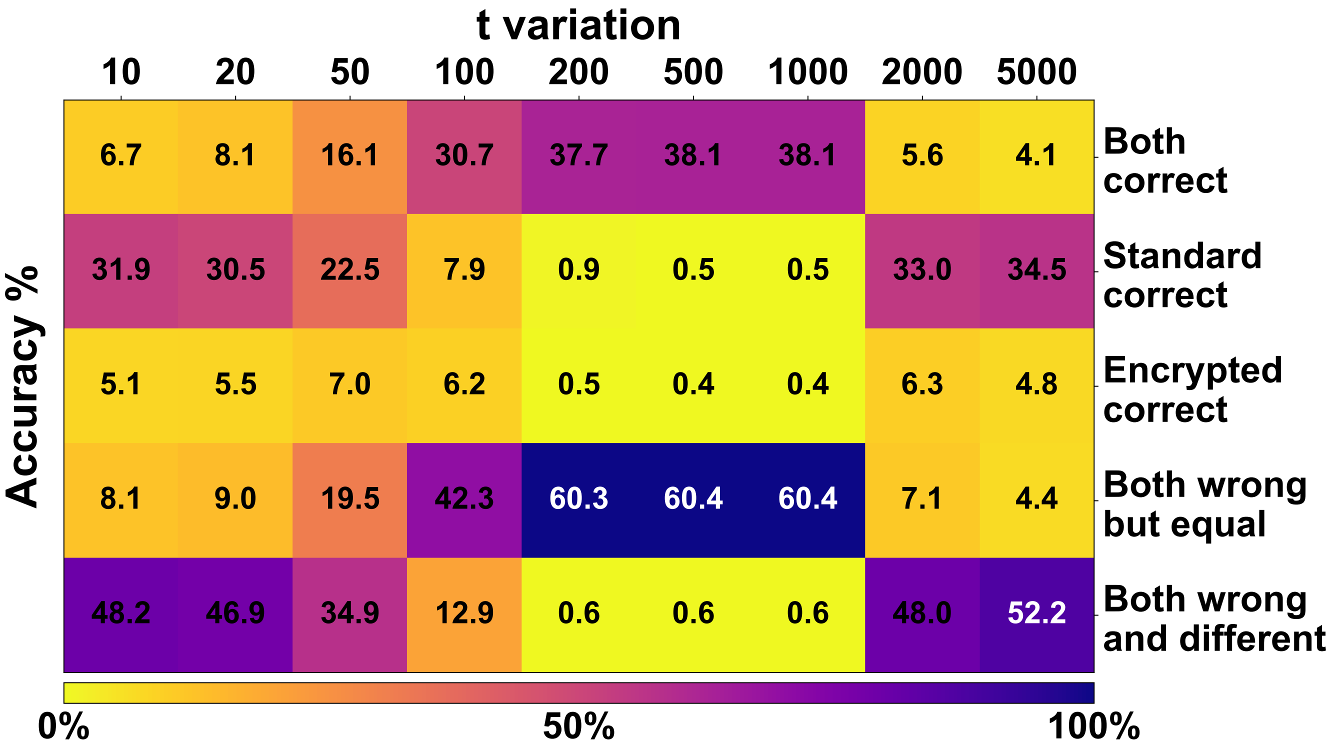

Figure S10: Comparison matrix for t variation and m set to 1024 for the CIFAR10 dataset on encrypted Spiking-LeNet5 model.

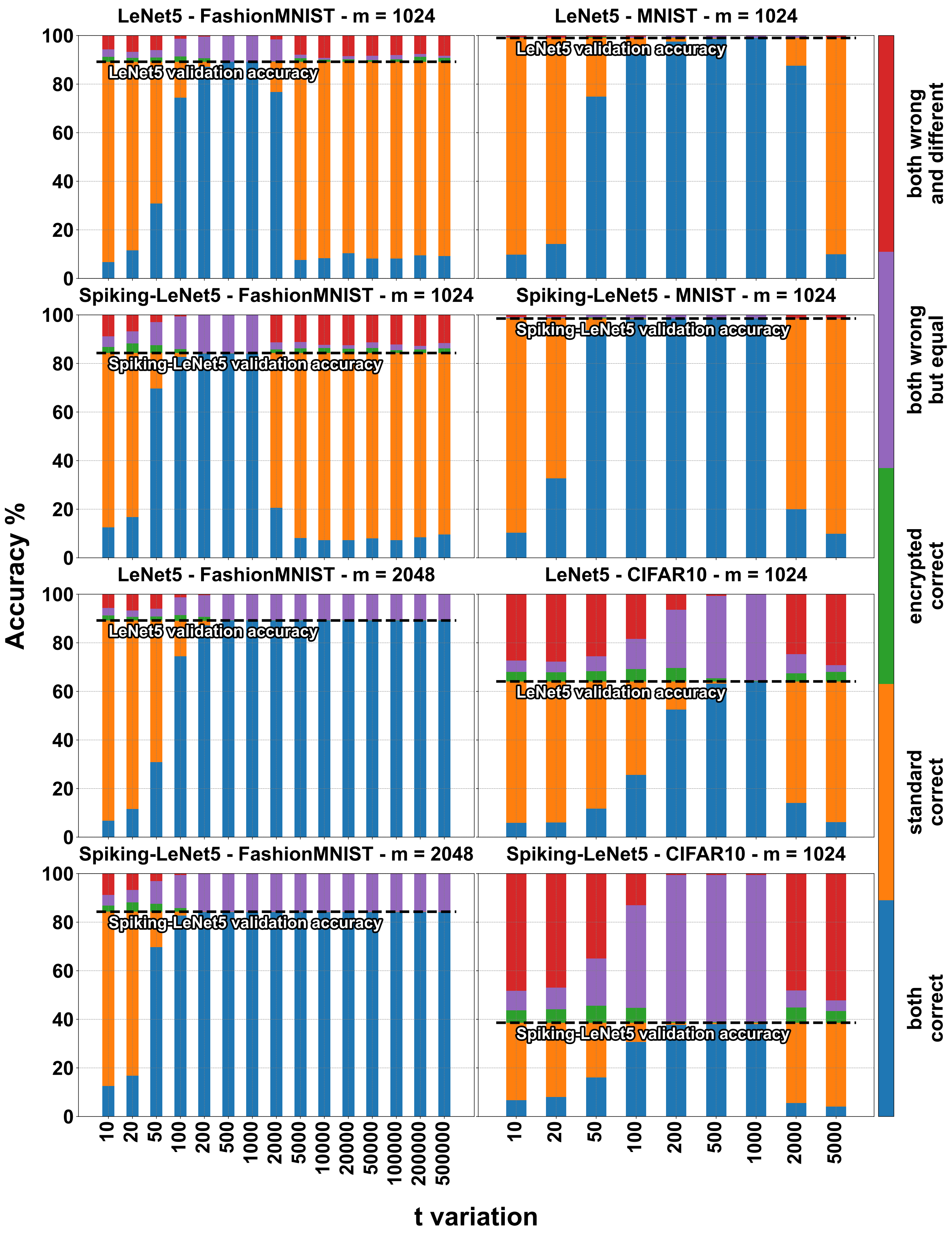

Figure S11: Stacked-bar comparison for all the datasets accuracy on encrypted LeNet5 and Spiking-LeNet5 models for t and m variation.

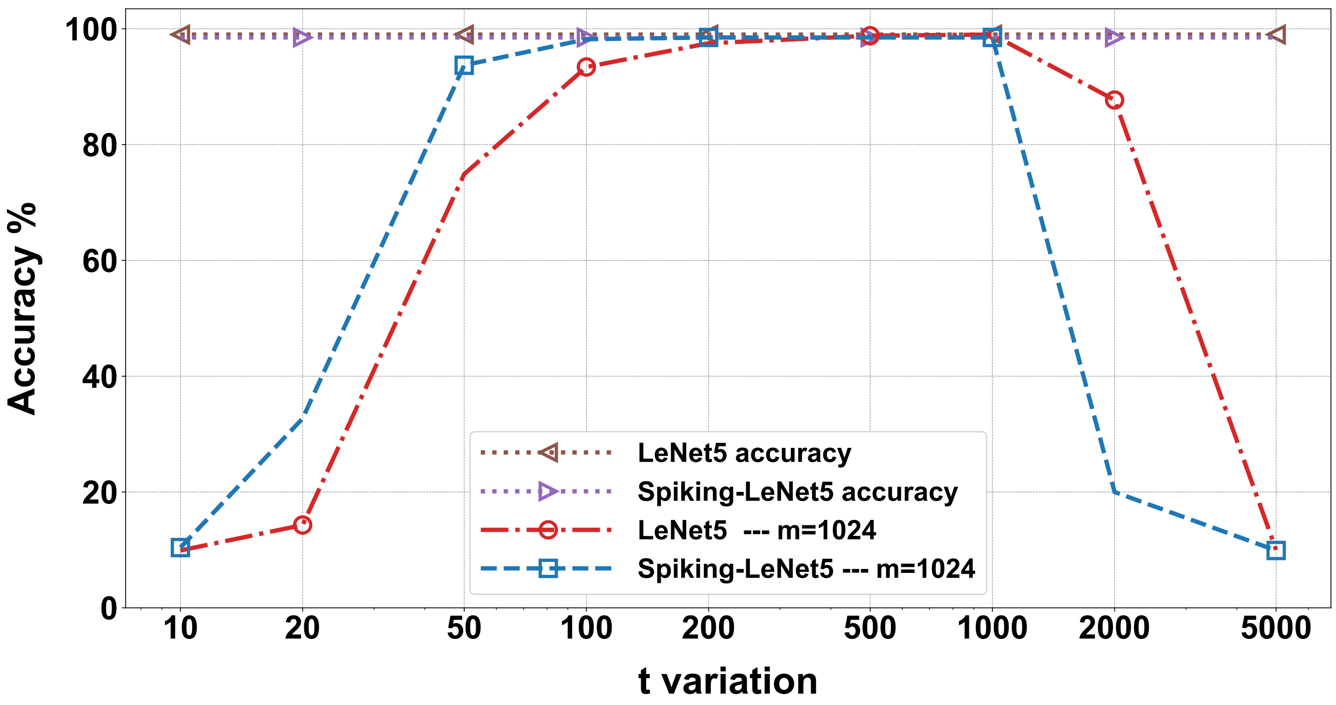

Figure S12: Comparison of MNIST accuracy between plaintext and encrypted versions of LeNet5 and Spiking-LeNet5 for t variations when both plaintext and encrypted versions classified correctly.

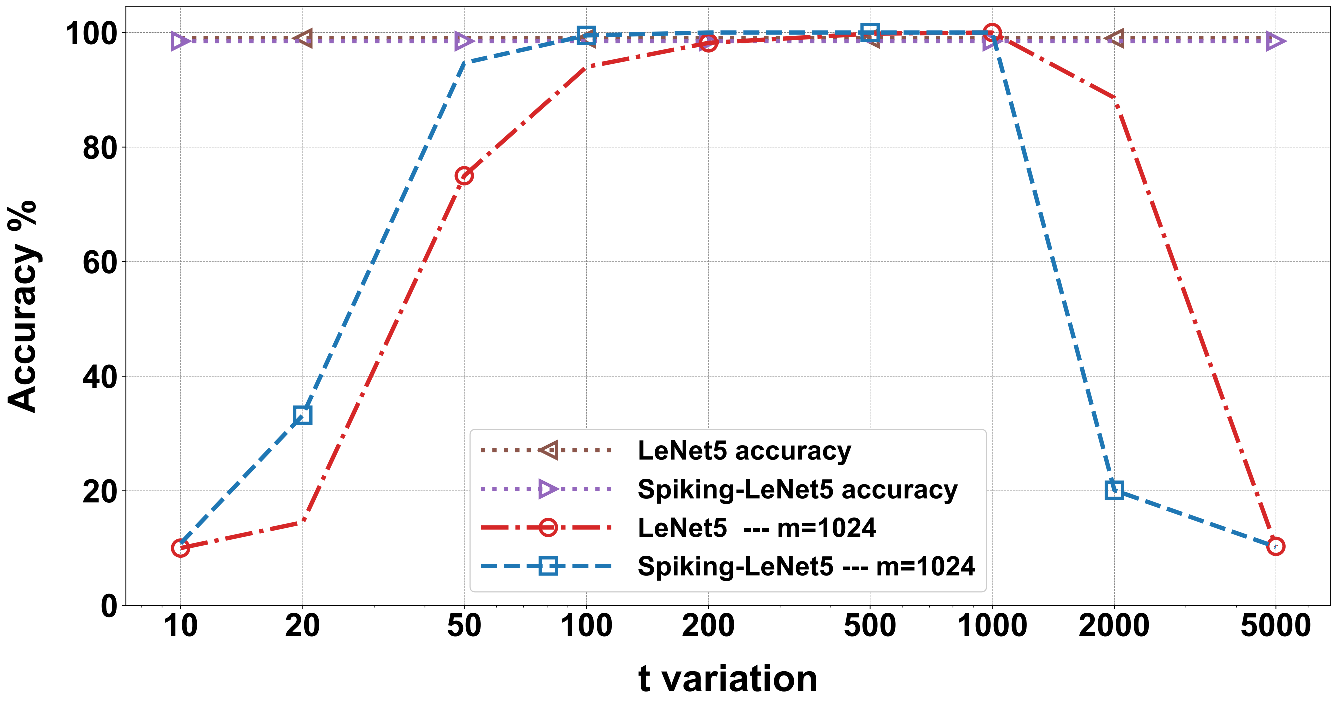

Figure S13: Comparison of MNIST accuracy between plaintext and encrypted versions of LeNet5 and Spiking-LeNet5 for t variations when both plaintext and encrypted versions coincide in both correct and incorrect classification.

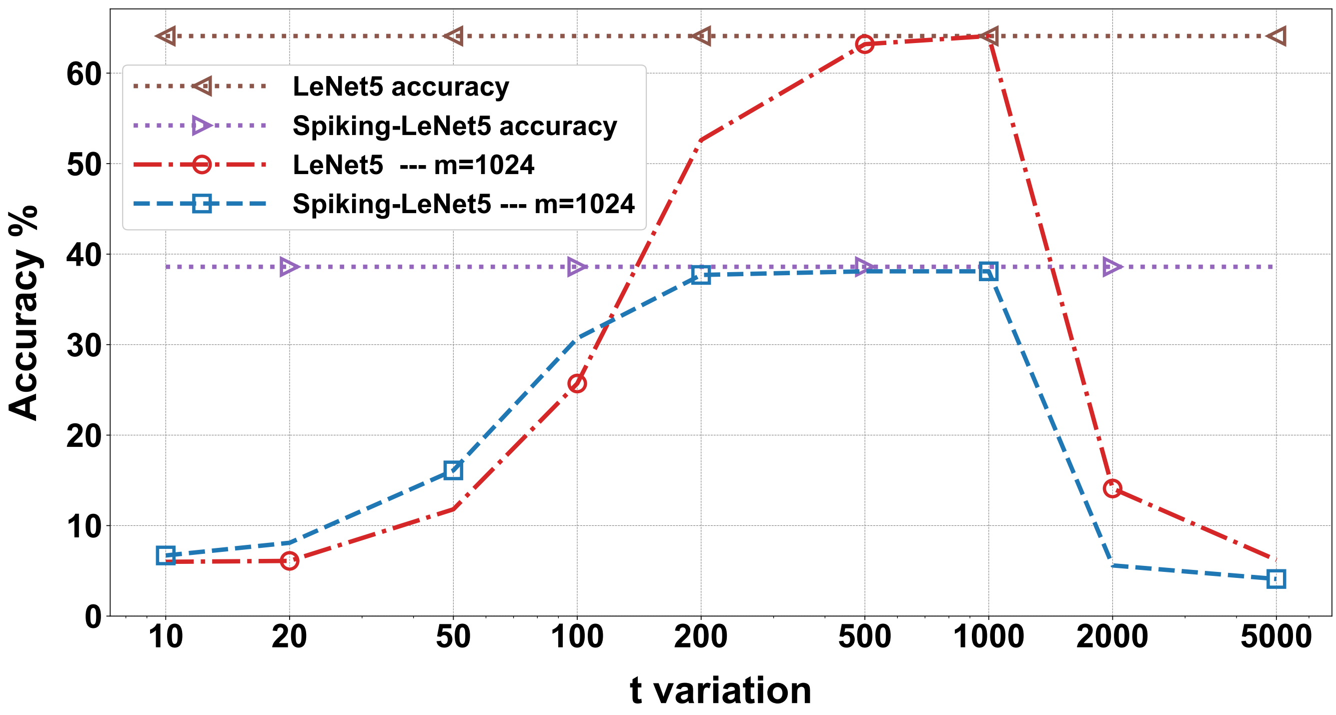

Figure S14: Comparison of CIFAR10 accuracy between plaintext and encrypted versions of LeNet5 and Spiking-LeNet5 for t variations when both plaintext and encrypted versions classified correctly.

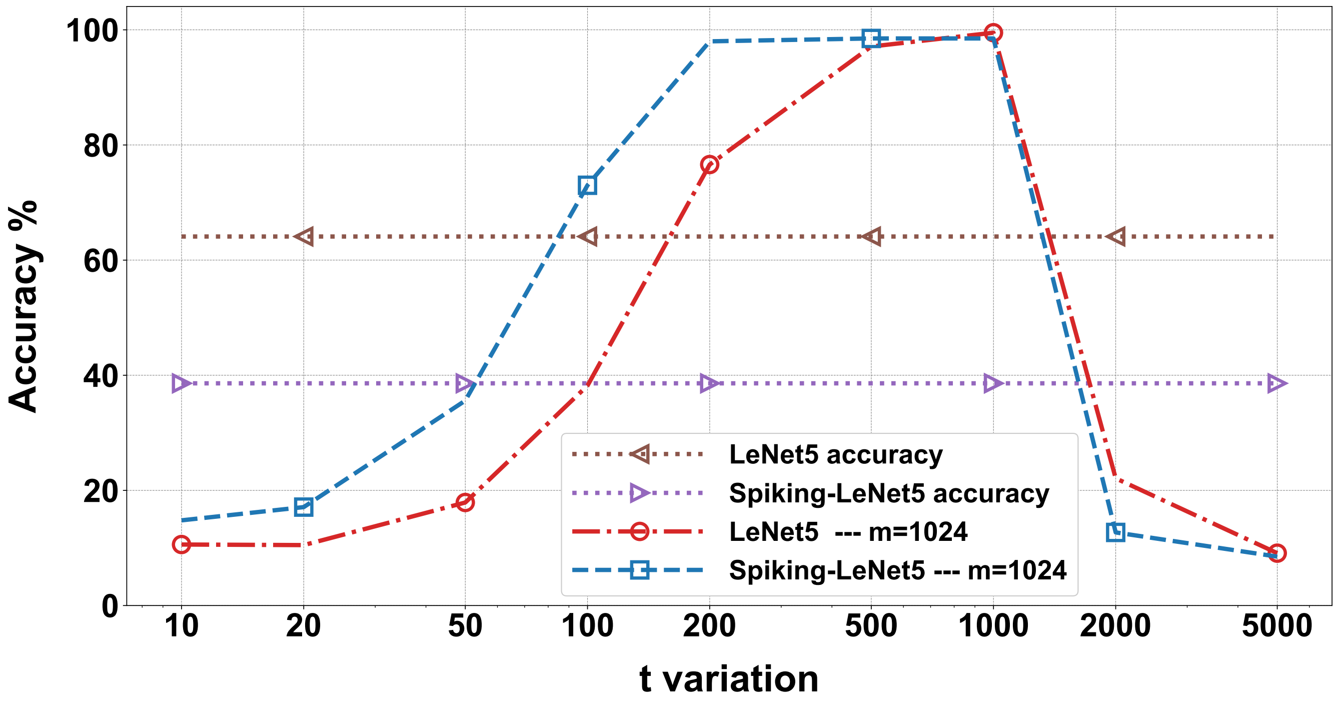

Figure S15: Comparison of CIFAR10 accuracy between plaintext and encrypted versions of LeNet5 and Spiking-LeNet5 for t variations when both plaintext and encrypted versions coincide in both correct and incorrect classification.

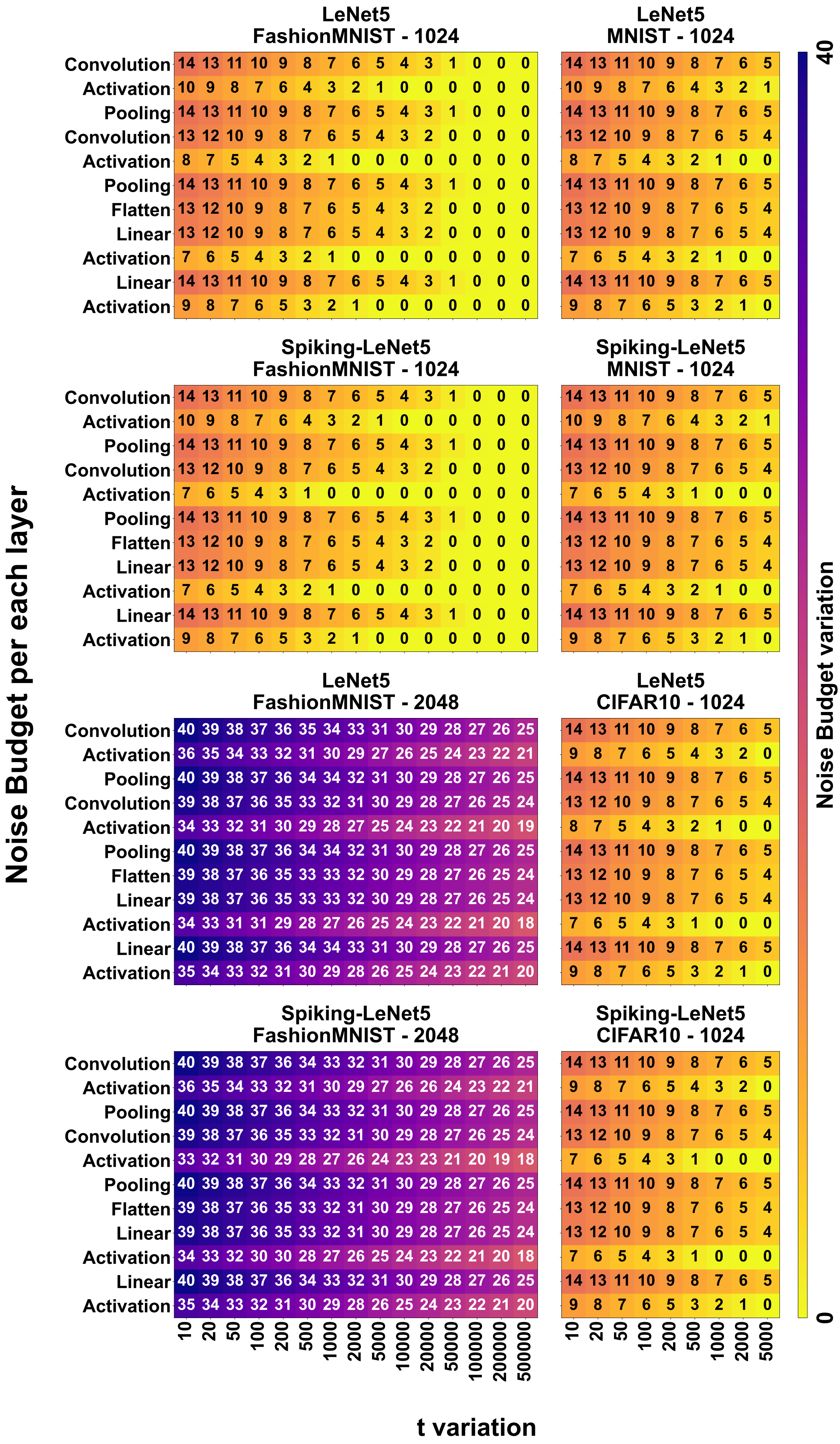

Figure S16: NB values for each model, dataset, m and t variation.

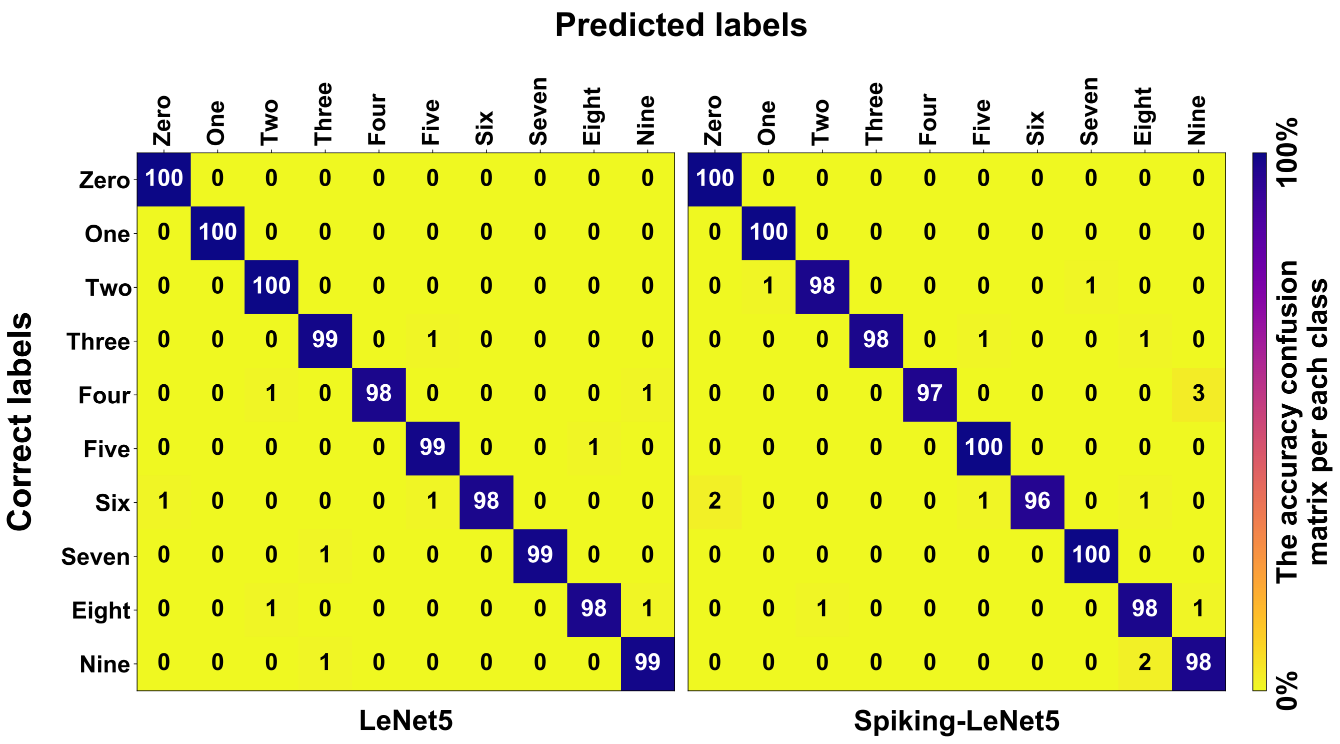

Figure S17: Plaintext confusion matrix for MNIST. The class 8 is the one that misleads the model the most.

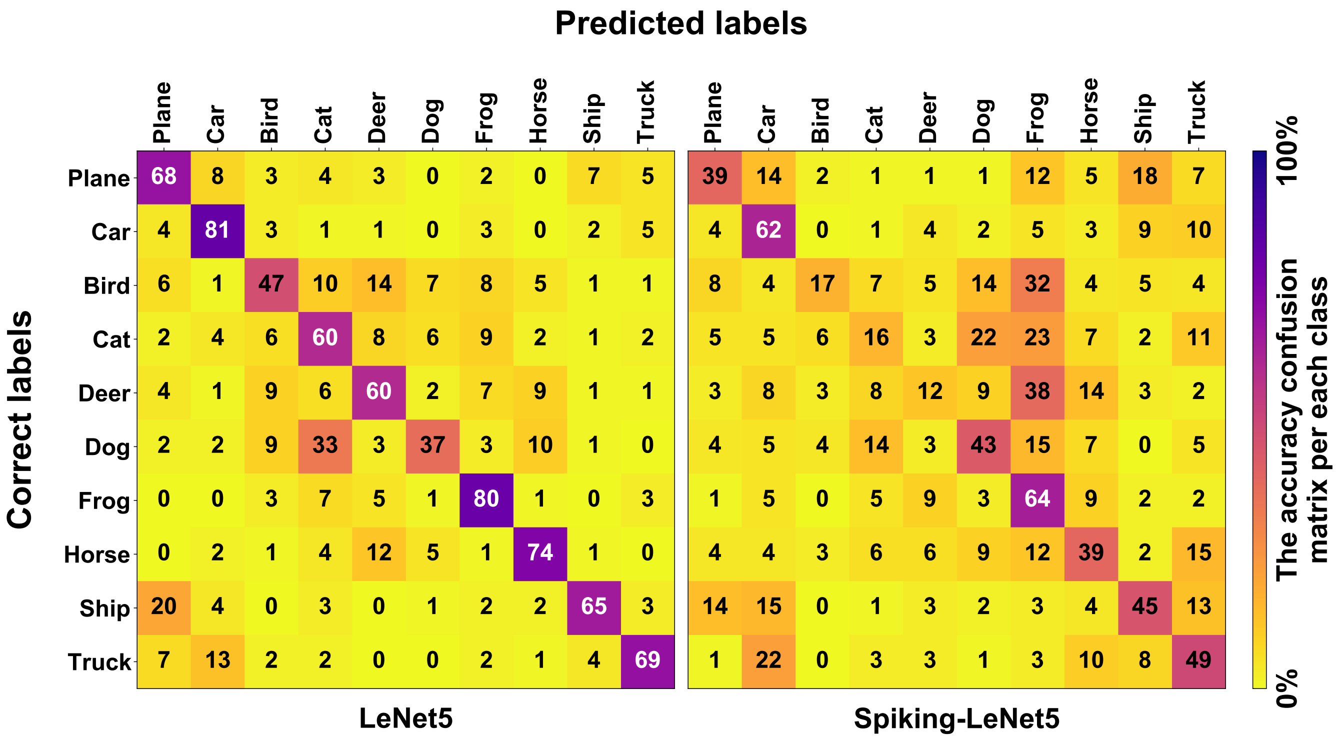

Figure S18: Plaintext confusion matrix for CIFAR10.

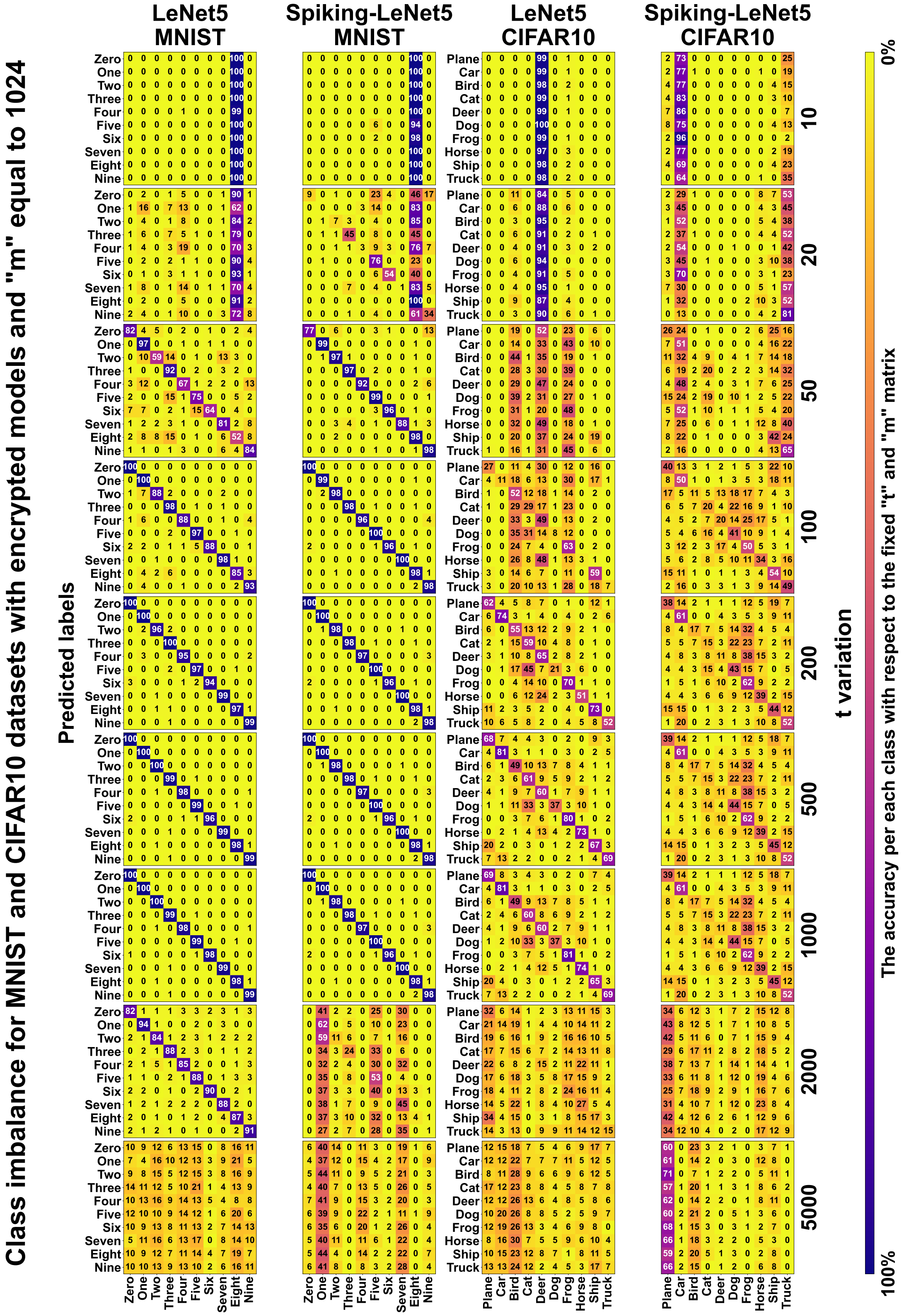

Figure S19: Encrypted confusion matrix for MNIST and CIFAR10 with t variation and m equal to 1024. It can be noticed that for low values of t, the results tend to concentrate on labels that resemble each other the most. Spiking-LeNet5 is less random than LeNet5 for low values of t.

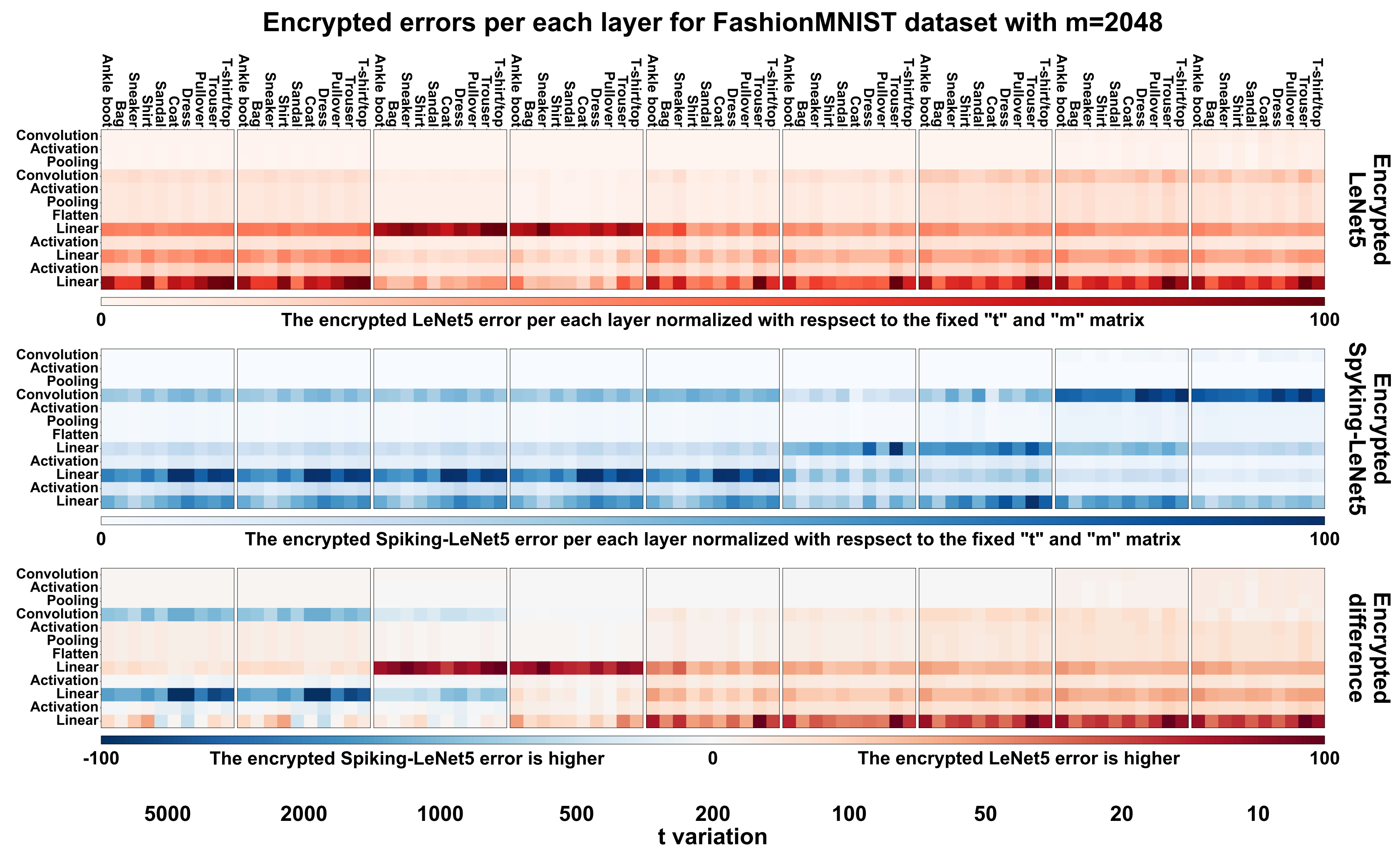

Figure S20: Errors layer-by-layer with FashionMNIST and $m = 2048$. The top **Red** strip represents the errors in the layers of the LeNet5, the **Blue** strip in the middle represents the errors in the layers of the Spiking-LeNet5. The last strip at the bottom represents the difference between the errors in the layers of LeNet-5 and Spiking-LeNet5. It can be noticed that the third strip is predominantly **Red**, indicating that Spiking-LeNet5 generally performs better.

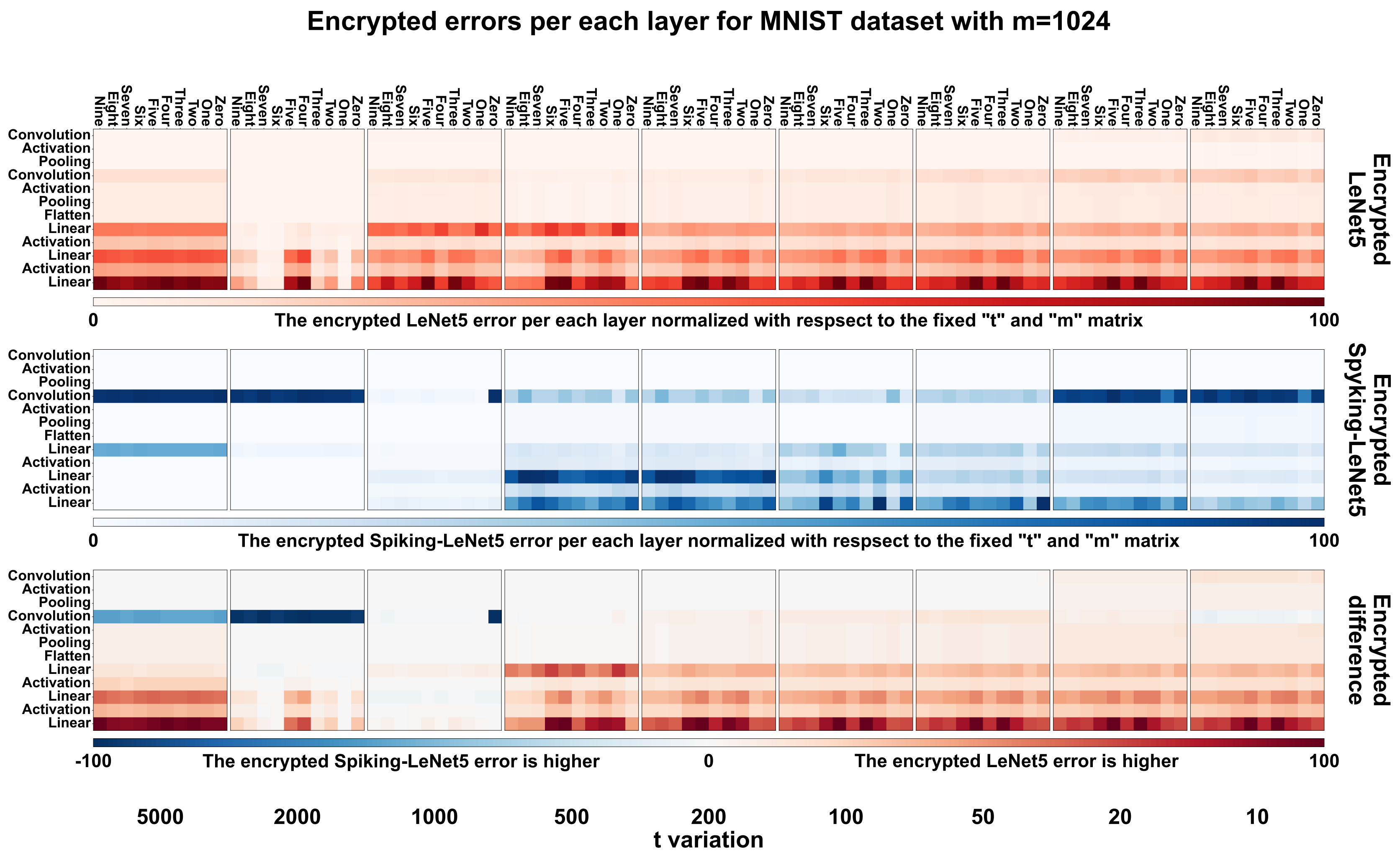

Figure S21: Errors layer-by-layer with MNIST and $m = 1024$. The top **Red** strip represents the errors in the layers of the LeNet5, the **Blue** strip in the middle represents the errors in the layers of the Spiking-LeNet5. The last strip at the bottom represents the difference between the errors in the layers of LeNet-5 and Spiking-LeNet5. It can be noticed that the third strip is predominantly **Red**, indicating that Spiking-LeNet5 generally performs better.

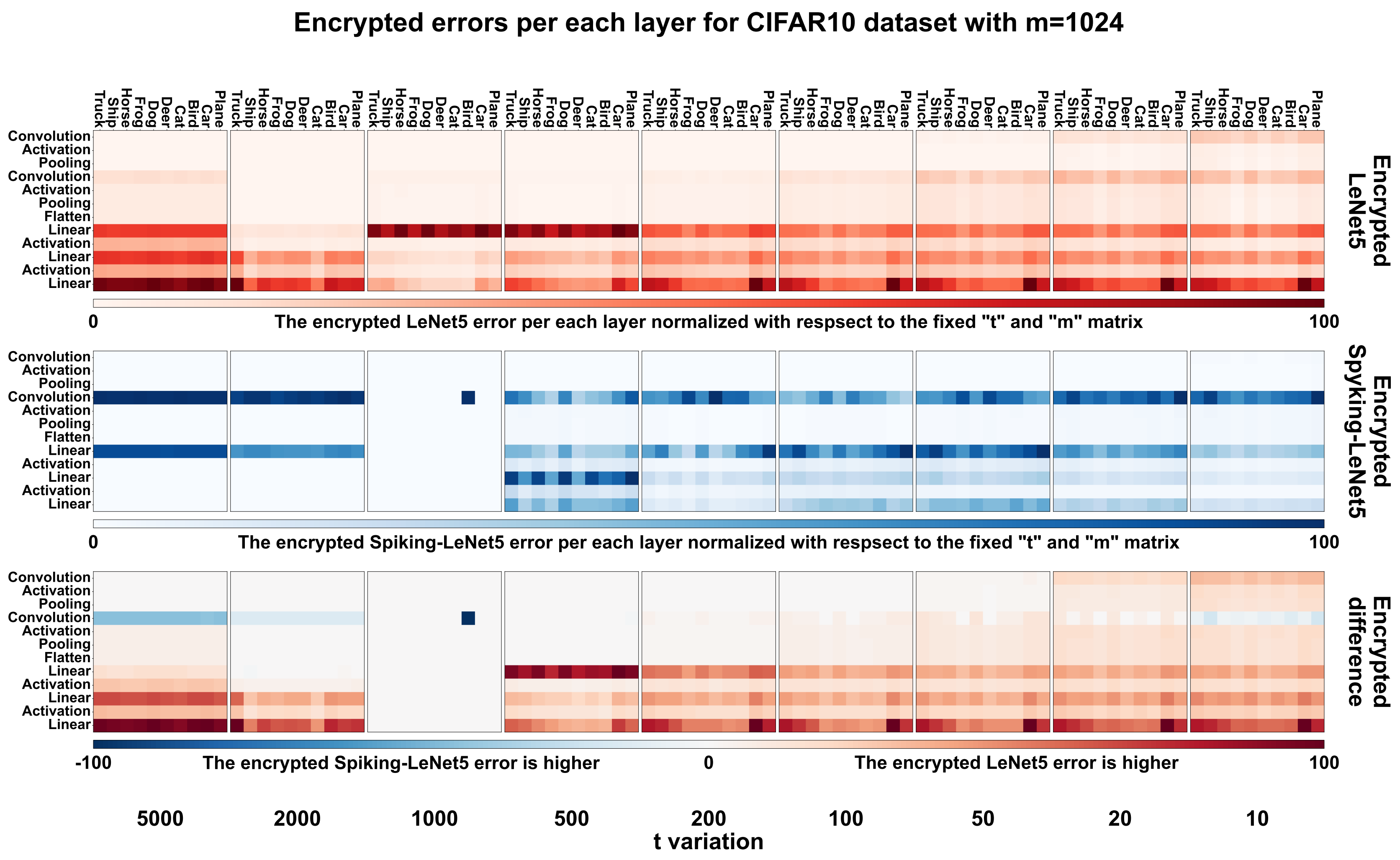

Figure S22: Errors layer-by-layer with CIFAR10 and m = 1024. The top **Red** strip represents the errors in the layers of the LeNet5, the **Blue** strip in the middle represents the errors in the layers of the Spiking-LeNet5. The last strip at the bottom represents the difference between the errors in the layers of LeNet-5 and Spiking-LeNet5. It can be noticed that the third strip is predominantly **Red**, indicating that Spiking-LeNet5 generally performs better.
